# Establishment of closed 35S ribosomal RNA gene chromatin in stationary *Saccharomyces cerevisiae* cells

**DOI:** 10.1093/nar/gkae838

**Published:** 2024-10-07

**Authors:** Virginia Babl, Philipp Girke, Sebastian Kruse, Sophia Pinz, Katharina Hannig, Christopher Schächner, Kristin Hergert, Manuel Wittner, Wolfgang Seufert, Philipp Milkereit, Herbert Tschochner, Joachim Griesenbeck

**Affiliations:** Regensburg Center of Biochemistry (RCB), Institut für Biochemie, Genetik und Mikrobiologie, Universität Regensburg, Lehrstühle Biochemie III und Genetik, Universitätsstr. 31, 93053 Regensburg, Germany; Regensburg Center of Biochemistry (RCB), Institut für Biochemie, Genetik und Mikrobiologie, Universität Regensburg, Lehrstühle Biochemie III und Genetik, Universitätsstr. 31, 93053 Regensburg, Germany; Regensburg Center of Biochemistry (RCB), Institut für Biochemie, Genetik und Mikrobiologie, Universität Regensburg, Lehrstühle Biochemie III und Genetik, Universitätsstr. 31, 93053 Regensburg, Germany; Regensburg Center of Biochemistry (RCB), Institut für Biochemie, Genetik und Mikrobiologie, Universität Regensburg, Lehrstühle Biochemie III und Genetik, Universitätsstr. 31, 93053 Regensburg, Germany; Regensburg Center of Biochemistry (RCB), Institut für Biochemie, Genetik und Mikrobiologie, Universität Regensburg, Lehrstühle Biochemie III und Genetik, Universitätsstr. 31, 93053 Regensburg, Germany; Regensburg Center of Biochemistry (RCB), Institut für Biochemie, Genetik und Mikrobiologie, Universität Regensburg, Lehrstühle Biochemie III und Genetik, Universitätsstr. 31, 93053 Regensburg, Germany; Regensburg Center of Biochemistry (RCB), Institut für Biochemie, Genetik und Mikrobiologie, Universität Regensburg, Lehrstühle Biochemie III und Genetik, Universitätsstr. 31, 93053 Regensburg, Germany; Regensburg Center of Biochemistry (RCB), Institut für Biochemie, Genetik und Mikrobiologie, Universität Regensburg, Lehrstühle Biochemie III und Genetik, Universitätsstr. 31, 93053 Regensburg, Germany; Regensburg Center of Biochemistry (RCB), Institut für Biochemie, Genetik und Mikrobiologie, Universität Regensburg, Lehrstühle Biochemie III und Genetik, Universitätsstr. 31, 93053 Regensburg, Germany; Regensburg Center of Biochemistry (RCB), Institut für Biochemie, Genetik und Mikrobiologie, Universität Regensburg, Lehrstühle Biochemie III und Genetik, Universitätsstr. 31, 93053 Regensburg, Germany; Regensburg Center of Biochemistry (RCB), Institut für Biochemie, Genetik und Mikrobiologie, Universität Regensburg, Lehrstühle Biochemie III und Genetik, Universitätsstr. 31, 93053 Regensburg, Germany; Regensburg Center of Biochemistry (RCB), Institut für Biochemie, Genetik und Mikrobiologie, Universität Regensburg, Lehrstühle Biochemie III und Genetik, Universitätsstr. 31, 93053 Regensburg, Germany

## Abstract

As a first step in eukaryotic ribosome biogenesis RNA polymerase (Pol) I synthesizes a large ribosomal RNA (rRNA) precursor from multicopy rRNA gene loci. This process is essential for cellular growth and regulated in response to the cell’s physiological state. rRNA gene transcription is downregulated upon growth to stationary phase in the yeast *Saccharomyces cerevisiae*. This reduction correlates with characteristic changes in rRNA gene chromatin structure from a transcriptionally active ‘open’ state to a non-transcribed ‘closed’ state. The conserved lysine deacetylase Rpd3 was shown to be required for this chromatin transition. We found that Rpd3 is needed for tight repression of Pol I transcription upon growth to stationary phase as a prerequisite for the establishment of the closed chromatin state. We provide evidence that Rpd3 regulates Pol I transcription by adjusting cellular levels of the Pol I preinitiation complex component core factor (CF). Importantly, our study identifies CF as the complex limiting the number of open rRNA genes in exponentially growing and stationary cells.

## Graphical Abstract

**Graphical Abstract:**
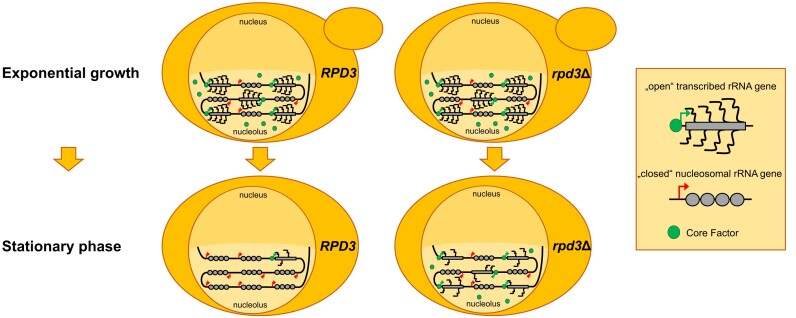

## Introduction

Eukaryotic transcription occurs in the context of chromatin, whose basic repetitive substructure is called the nucleosome (reviewed in (1)). Nucleosomes, consisting of 146 bp of DNA wrapped around a histone octamer, are needed for DNA compaction in the nucleus and restrict access of the transcription machinery to genetic elements contributing to the regulation of RNA synthesis. In good agreement, changes in local chromatin structure are observed upon gene activation (reviewed in (2)). These changes include alterations in the occupancy and position of nucleosomes as well as posttranslational modifications of histone molecules. Accordingly, enzymatic activities required for nucleosome remodeling or the attachment or removal of chemical groups to histone proteins have been identified as regulators of transcription.

The conserved lysine deacetylase Rpd3 (HDAC1 in multicellular plants and metazoans) was identified as a transcriptional regulator in *Saccharomyces cerevisiae* (hereafter referred to as yeast) (reviewed in (3–6)). Its enzymatic activity was uncovered after biochemical fractionation from mammalian cells (7). Rpd3 de-acetylates histones and exerts specific functions in the context of several multi-protein complexes. Besides its role in regulating the transcription of RNA polymerase (Pol) II-dependent genes, a distinct function for Rpd3 has been described in regulating chromatin structure at yeast ribosomal RNA (rRNA) gene loci transcribed by Pol I (8,9). Independently, Rpd3 was identified as a repressor of Pol I transcription in a genetic screen (10).

**Figure 1.**
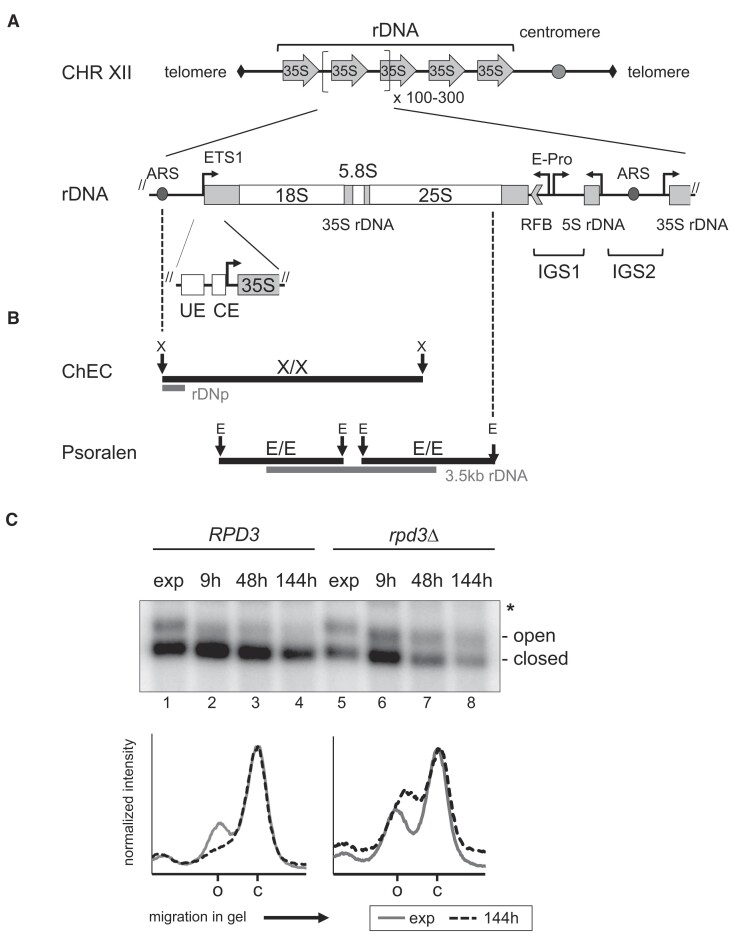
(**A**) Schematic representation of the rDNA locus of *Saccharomyces cerevisiae*. The yeast rDNA locus located on chromosome XII (CHR XII) consisting of 100–300 transcription units arranged in a head-to-tail orientation is shown at the top. In the middle, an rDNA transcription unit with genetic elements is depicted. The 5S rDNA transcribed by Pol III is separated by intergenic spacers (IGS1/2) from two 35S rRNA gene copies. IGS1 contains a RFB, a bidirectional, Pol II-dependent expansion promoter (E-Pro) and an ARS found within IGS2. The 35S rDNA is transcribed by Pol I, producing a large precursor which is processed to the mature 18S, 5.8S and 25S rRNAs. The 5′ external transcribed spacer (ETS1) is indicated. The 35S rDNA promoter composed of the UE and CE is shown at the bottom. Arrows point in the direction of transcription. (**B**) Fragments of the rDNA analyzed in ChEC and psoralen photo-crosslinking experiments in this study: X, E, XcmI, EcoRI restriction sites. Names of radioactively labeled probes and their hybridization sites (gray bars) in Southern blot analysis are depicted below the fragments (details are provided in Supplementary Table S5). (**C**) Deletion of *RPD3* prevents growth phase-dependent closing of 35S rRNA genes. *RPD3* and *rpd3*Δ strains NOY505 and y2919 were grown in YPAD containing 1% glucose at 30°C to exponential phase (exp), and for another 9, 48 and 144 h, before samples were subjected to psoralen photo-crosslinking (Supplementary Figure S1A). DNA was isolated, cleaved with EcoRI and analyzed in a Southern blot hybridized with probe ‘3.5 kb rDNA’ (Figure 1B and Supplementary Table S5). Autoradiograms show the results for the 2 kb 18S rDNA fragment. The positions of strongly psoralen-crosslinked fragments derived from ‘open’ rRNA genes and of weakly psoralen-crosslinked ‘closed’ rRNA genes are indicated on the right. The position of a band resulting from incomplete digestion of psoralen-crosslinked DNA is labeled by an asterisk. Graphs at the bottom show analyses of the radioactivity profile in the lanes with samples ‘exp’ (gray lines) and after 144 h of cultivation (dotted black lines). Each profile was normalized to the maximum intensity of the fragment derived from closed rRNA genes and plotted against the migration in the gel. The positions of the peaks corresponding to the fragments derived from open (o) and closed (c) rRNA genes are labeled on the *x*-axis of the graphs. A representative result of four biological replicates is shown.

In eukaryotic cells, the Pol I transcription machinery is dedicated to synthesizing the large precursor RNA for three out of the four mature rRNAs forming the nucleic acid scaffold of ribosomes (reviewed in (11,12)). rRNA transcription occurs together with the early steps of ribosome biogenesis in a membrane-less nuclear compartment, the nucleolus. Large amounts of ribosomes need to be synthesized in an actively dividing eukaryotic cell and Pol I transcription accounts for the major part of cellular RNA synthesis during exponential growth (13). This high transcriptional output is achieved by two means: a high transcription rate at individual rRNA genes and the presence of multiple rRNA gene copies in the genome. In yeast rRNA gene transcription occurs at 150–300 tandemly repeated transcription units on chromosome XII collectively called the ribosomal DNA (rDNA) locus (Figure 1A, top). Each of these transcription units contains the 5S rRNA gene transcribed by Pol III, flanked by 35S rRNA genes transcribed by Pol I to yield the precursor for the mature 18S, 5.8S and 25S rRNAs (Figure 1A, middle). The Pol I and Pol III transcribed genes are separated by two intergenic spacer sequences (IGS1/2), which bear other functional elements like a replication fork barrier (RFB), a Pol II-dependent expansion promoter (E-pro) and an autonomous replication sequence (ARS). Pol I transcription is controlled by the bipartite 35S rDNA promoter, containing the upstream element (UE) and the core element (CE) (14,15) (Figure 1A, bottom). These elements are bound by upstream activation factor (UAF, composed of Uaf30, Rrn5, Rrn9, Rrn10, histones H3 and H4) (16–18) and core factor (CF, composed of Rrn6, Rrn7, Rrn11) (19–21). To initiate transcription, UAF binds to the UE and recruits the TATA-binding protein (TBP, Spt15 in yeast) and CF (22,23). Whereas CF is required for Pol I initiation *in vitro*, UAF is not needed for basal transcription in the purified system but can stimulate transcription (24). *In vivo*, however, Pol I transcription of rRNA genes is abolished or at least severely impaired in the absence of individual UAF subunits (17,23,25). Recruitment of initiation competent Pol I in complex with the conserved Pol I specific transcription factor Rrn3 completes preinitiation complex (PIC) formation (22,23,26). After promoter clearance, Rrn3 dissociates from the Pol (26,27). By a so far unknown mechanism, the high-mobility group (HMG)-box protein Hmo1 associates with Pol I transcribed, nucleosome-depleted 35S rDNA (28–31).

Although efficient rRNA synthesis is needed for cellular growth, only a subpopulation of the multicopy 35S rRNA genes is transcribed in individual proliferating cells (32,33). The transcriptional status of 35S rRNA genes correlates with specific chromatin states. Transcribed genes reside in a nucleosome-depleted ‘open’ chromatin state, whereas non-transcribed genes reside in a nucleosomal ‘closed’ chromatin state (reviewed in (34–37)). Chromatin transitions of 35S rRNA genes were observed in various situations. In exponentially growing cells genes switch between the two chromatin states as a consequence of Pol I transcription-dependent chromatin opening and replication-dependent chromatin closing (38). When replication-dependent nucleosome assembly is prevented, most of the genes eventually reach the open chromatin state. Once established, binding of the HMG-box protein Hmo1 to rRNA genes may stabilize the open gene chromatin state even in the absence of Pol I transcription (38). Thus, Hmo1 presumably interferes with replication-independent nucleosome deposition. Closing of 35S rRNA gene chromatin has been observed when nutrients become limiting, e.g., when yeast cells grow to stationary phase (9,33,39).

Upon growth to the stationary phase, Pol I occupancy at 35S rRNA genes is substantially reduced as soon as cells exit exponential growth. A diminished target of rapamycin (TOR) signaling has been implicated in this process (40). The reduction in Pol I association with 35S rRNA genes in this situation is thought to be regulated at the level of transcription initiation affecting the formation of the Rrn3-Pol I complex (26,40,41). The downregulation of Pol I transcription during growth to the stationary phase correlates with the closing of rRNA gene chromatin (9,33,39). Interestingly, it was found that the latter requires the activity of the lysine-deacetylase Rpd3 (9). Upon deletion of *RPD3*, the major fraction of 35S rRNA genes remained in the open chromatin state in stationary cells albeit Pol I transcription was still significantly downregulated. However, residual Pol I transcription measured in transcription run-on experiments was higher in stationary *rpd3*Δ cells than in *RPD3* cells. Taken together, it was hypothesized that Pol I transcription may be repressed by two independent mechanisms upon growth to stationary phase: TOR-signaling dependent reduction of transcription initiation and Rpd3-dependent chromatin alterations limiting the number of open 35S rRNA genes (9,42). Since there is evidence that an Rpd3 core complex may act as a chromatin stabilization module (43), the maintenance of the open chromatin state in *rpd3*Δ cells has been mechanistically linked to impaired nucleosome retention on 35S rRNA genes (8).

Here, we found that the maintenance of open 35S rRNA gene chromatin in stationary *rpd3*Δ cells is likely the consequence of low levels of Pol I transcription initiating at multiple 35S rDNA promoters which are poised for transcription. Once established, the open chromatin state appears to be stabilized by Hmo1. Our results suggest, that Rpd3 is required for tight repression of Pol I transcription in the stationary phase by limiting the cellular levels of CF components and thus PIC formation.

## Materials and methods

### Oligonucleotides, plasmids and yeast strains

Unless noted otherwise, standard techniques were used for cloning of plasmids and transformation of yeast cells (44–46). Information about oligonucleotides, plasmids and yeast strains used in this study can be found in Supplementary Tables S1–S3 including details on plasmid and strain construction. Plasmid sequences are available upon request. For the individual experiments in this study, yeast cells were grown for the indicated times at the indicated temperatures in either YPD (2% [w/v] peptone, 1% [w/v] yeast extract, 2% [w/v] glucose), YPAD (YPD including 100 mg/l adenine), YPAD+ (YPAD including 0.2 g/l tryptophan, 10 mM KH_2_PO_4_), or 2xYPAR (4% [w/v] peptone, 2% [w/v] yeast extract, 4% [w/v] raffinose) unless noted otherwise. Yeast cell growth to the stationary phase was followed by measuring the optical density of the culture at 600 nm (OD600). OD600 values for most of the individual experiments are available as Supplementary Data Set SD1.

### Gel electrophoresis and blot analysis

Whole-cell protein extracts were prepared as described (47), and sodium dodecyl sulfate-polyacrylamide gel electrophoresis (SDS-PAGE) and western blot analyses were performed according to standard procedures (48,49). Gels were transferred to nitrocellulose membranes for subsequent immuno-detection. Protein transfer to membranes was verified by staining with Ponceau S. Supplementary Table S4 contains a complete list of antibodies used for detection. Antibodies coupled to horseradish peroxidase (HRP) were visualized using BM Chemiluminescence Western Blotting Substrate (POD, Roche) and a LAS-3000 Chemiluminescence Imager (Fujifilm). Fluorescently labeled antibodies (LI-COR Biosciences) were visualized with an Odyssey infrared imaging system (LI-COR Biosciences).

**Figure 2.**
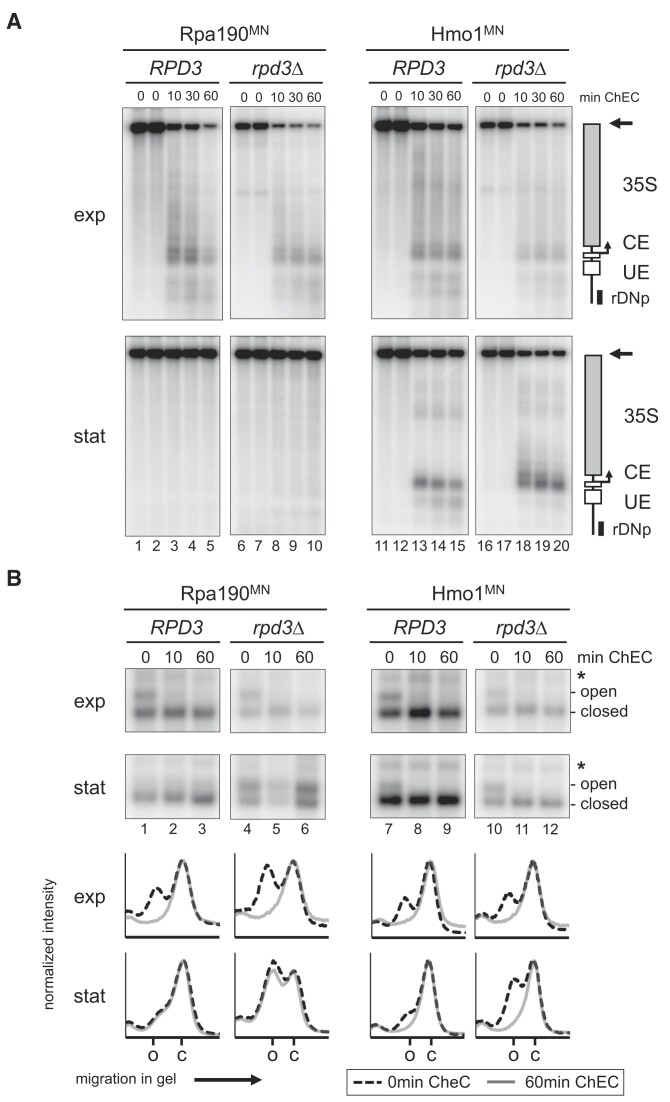
Open rRNA genes in stationary rpd3Δ cells are largely devoid of Pol I and are bound by Hmo1. *RPD3* or *rpd3*Δ strains y1717, y2256, y1761 and y2257, expressing either Rpa190^MN^ or Hmo1^MN^ were grown in YPAD at 30°C to exponential phase (exp) or for another 144h to stationary phase (stat). Samples were withdrawn at each time point. (**A**) Samples were either mock treated (0 min ChEC) or subjected to ChEC experiments for the times indicated on top, DNA was isolated, digested with XcmI, and analyzed in a Southern blot hybridized with probe ‘rDNp’ (Figure 1B, Supplementary Table S5 and Supplementary Figure S1B). Autoradiograms are shown. A map of the XcmI fragment under investigation is depicted on the right (genetic elements as explained in Figure 1A). An arrow labels the full-length XcmI fragment. (**B**) Selected ChEC samples were subjected to psoralen photo-crosslinking as described in the legend of Figure 1C (Supplementary Figure S1C). The positions of psoralen-crosslinked fragments derived from 'open' rRNA genes and from ‘closed’ rRNA genes are indicated on the right. The position of a band resulting from incomplete digestion of psoralen-crosslinked DNA is labeled by an asterisk. Graphs at the bottom show analyses of the radioactivity profile in the lanes with samples of the 0 min (dotted black lines) and 60 min ChEC time points (gray lines). The positions of the peaks corresponding to the fragments derived from open (o) and closed (c) rRNA genes are labeled on the *x*-axis of the graphs at the bottom. Representative results of two (Rpa190^MN^ expressing cells) and three (Hmo1^MN^ expressing cells) biological replicates are shown.

Agarose gel electrophoresis and Southern blot analysis were performed as described (45), with the exception that the transfer of nucleic acids onto nylon membranes (Positive Membrane, Qbiogen) by capillary transfer was performed with 1 M ammonium acetate (50). Supplementary Table S5 contains a list of templates used for the synthesis of radiolabeled hybridization probes generated using the RadPrime DNA labeling system (GE Healthcare Life Sciences). Hybridization sites for the respective radioactively labeled probes are shown in Figure 1B. In psoralen photo-crosslinking experiments shown in Figures 1, 2, 6 and Supplementary Figures S2 and S6, only one of two 2- and 2.8-kb EcoRI fragments spanning almost the entire 35S rDNA region transcribed by Pol I is shown, as indicated in the figure legends (Figure 1B depicts a schematic representation of the respective DNA fragments). In all cases, both fragments behaved identically. Radioactive blots were exposed to imaging plates, which were scanned with an FLA-3000 (Fujifilm) or an FLA-9500 (GE Healthcare Life Sciences) system. The image data of chromatin endogenous cleavage (ChEC) and ChEC-psoralen experiments were processed with Multi Gauge software (Fujifilm) using the ‘profile’ module. Raw data were exported into Microsoft Excel. For the depiction of the profiles, the radioactivity intensities were normalized to the maximum intensity in each lane. The normalized intensities for selected lanes were then plotted against the distance of migration in the gel. To calculate the percentage of degradation of the full-length XcmI fragment in ChEC experiments, the background-corrected integrated radioactive intensity of cleavage products was divided by the sum of the integrated radioactive intensities of cleavage products and the integrated radioactive intensity of the full-length XcmI fragment for each lane.

### Chromatin endogenous cleavage (ChEC), psoralen photo-crosslinking and chromatin immunoprecipitation (ChIP)

For ChEC(-psoralen photo-crosslinking) and ChIP experiments, we employed yeast strains expressing different genes as fusion proteins with a C-terminal micrococcus nuclease (MN) followed by a triple hemagglutinin (HA)-tag and a C-terminal tandem affinity purification (TAP)-tag, respectively, from their endogenous genomic location. These strains were grown under the conditions indicated. ChEC and psoralen photo-crosslinking analyses were performed as previously described (31,51,52) (Supplementary Figure S1). All DNA samples were digested with either XcmI or EcoRI before agarose gel electrophoresis and Southern blot analysis (Figure 1B depicts a schematic representation of DNA fragments analyzed in this study; Supplementary Table S5 summarizes templates used for the synthesis of radioactively labeled probes for membrane hybridization). ChIP experiments were performed as described (53), except that Immunoglobulin G (IgG) coupled to sepharose beads (GE Healthcare) were used for the precipitation of TAP-tagged proteins. Relative DNA amounts present in input (in) and immunopreciptiation (ip) were determined by quantitative PCR using SYBR green I dye (Roche) for DNA detection with a Rotor-Gene Q system (Qiagen). Cycle threshold (CT) values and amplification efficiency (*E*) for a defined primer pair for all input (CTin) and ChIP samples (CTip) of an experimental dataset were determined with the Rotor-Gene Q software using the comparative quantification module. The CT value correlates with the Rotor-Gene software ‘take-off’ value. For each primer pair, the efficiency (*E*) is calculated as an average from the fluorescence measurements of each sample upon exponential amplification (54). The percentage of co-precipitated DNA was determined by the formula % ChIP = ((1 + E)^CTip^*DF/(1 + E)^CTin^)*100 (DF: the quotient of the dilution factors of input and ChIP samples). Average and standard deviation errors for ChIP are derived from three independent ChIP experiments, each analyzed in triplicate quantitative (q)PCRs. The original qPCR data, including CT values and efficiencies, amplification curves, melting curve analysis and mathematical operations are available as Supplementary Data Set SD2.

### RNA isolation and reverse transcriptase (RT) qPCR analysis

RNA was isolated as described (55). Reverse transcription was performed with Superscript III (Thermo Fisher) or the QuantiTect Reverse Transcription Kit (QIAGEN), according to the instructions of the manufacturer. qPCR analysis was performed as described for ChIP experiments. Relative 35S RNA cDNA amounts were calculated by normalizing 35S rRNA cDNA amounts to 5S rRNA cDNA amounts for each individual sample using the formula 35S cDNA/5S cDNA = (1 + E_5S_)^CT^_5S_/(1 + E_35S_)^CT^_35S_. E_5S/35S_ and CT_5S/35S_ are the efficiencies of PCR amplification and CT values obtained with primer pairs detecting 5S cDNA and 35S cDNA, respectively. Relative cDNA amounts of mRNAs of a gene of interest (GOI: *RRN6*, *RRN7*, *RRN11*, *UAF30*) were calculated by normalizing the respective cDNA amounts to the cDNA amounts of *PDC1* mRNA in each individual sample using the formula GOI cDNA/*PDC1* cDNA = (1 + E_PDC1_)^CT^_PDC1_/(1 + E_GOI_)^CT^_GOI._ E_PDC1/GOI_ and CT_PDC1/GOI_ are the efficiencies of PCR amplification and CT values obtained with primer pairs detecting *PDC1* cDNA and GOI cDNA, respectively. Average and standard deviation errors for relative 35S rRNA cDNA amounts are derived from two independent biological replicates, each analyzed in duplicate or triplicate qPCRs. For relative *RRN6*, *RRN7*, *RRN11* and *UAF30*cDNA levels two independent clones were analyzed in triplicate qPCRs. The original qPCR data, including CT values and efficiencies, amplification curves, melting curve analysis, and mathematical operations are available as Supplementary Data Set SD2.

### Live cell imaging

Cells were grown in YPAD+ overnight at 30°C. Cells in the exponential growth phase were harvested the next day, resuspended in water to remove YPAD+, pipetted on cover slides and covered with an agarose block containing synthetic complete medium (2% [w/v] glucose, yeast nitrogen base, amino acids, nucleobases) for imaging with an inverted microscope. The remaining cells were incubated for an additional 129 h at 30°C, harvested, resuspended in water to remove YPAD+, pipetted on cover slides and covered with an agarose block devoid of nutrients. Imaging was done with an Axio Observer.Z1 (Carl Zeiss) in combination with a CSU-X1 spinning disk unit (Yokogawa), a Plan Apochromat 63× /1.40 oil objective, an AxioCam MRm (Carl Zeiss) for detection and ZEN software (Carl Zeiss). Twelve z-slices with a distance of 500 nm were acquired. mCherry was excited with a 561 nm laser, and green fluorescent protein (GFP) was excited with a 488 nm laser. Cells were imaged at room temperature (22–24°C) immediately after sample preparation.

All image processing and quantifications were done with ImageJ (National Institutes of Health, Bethesda, MD (56)). Differential interference contrast (DIC) micrographs show single z-slices, and fluorescence channel micrographs show maximum intensity projections. Scale bars equal 5 μm. In merge images, the Hmo1^3mCherry^ signal is depicted in red, and GFP signals are depicted in cyan. For quantification, the background determined from regions outside cells was subtracted from images. The plugin 3D object counter (57) was used to determine the overall Rpa190^GFP^ signal within the thresholded signal of Hmo1^3mCherry^ (integrated density, redirect function). All quantifications were done with unprojected z-stacks. The intensity threshold was set to 50 and the size threshold was set to 30 to efficiently exclude regions showing background fluorescence when comparing exponentially growing and stationary *RPD3* and *rpd3*Δ cells. UAF and CF components showed signal intensities very close to the background in stationary cells and higher background in general due to the high laser intensity needed to detect those lowly abundant factors. Therefore, reliable measurements of the integrated densities of UAF and CF components in stationary cells were not possible.

Cell categorization was also done with unprojected z-stacks. ‘Colocalization’ was assigned when the major portion of the nucleolar GFP signal overlapped with Hmo1^3mCherry^. ‘Adjacent’ was assigned when the major portion of the nucleolar GFP signal did not overlap with but was directly adjacent to Hmo1^3mCherry^. ‘No nucleolar GFP’ was assigned when no GFP signal above the background could be seen within or directly adjacent to the Hmo1^3mCherry^ signal. ‘Lysed’ was assigned according to the disintegrated appearance in the DIC channel. Living cells lacking the Hmo1^3mCherry^ signal were excluded from analyses (one in 400 cells).

### Graphs and statistics

Box plots were done with the software Origin (OriginLab Corporation) and are specified as follows: the central box spans the first and third quartile (Q1 and Q3, respectively), the line inside the box represents the median (Q2), and a square represents the mean. Whiskers extending from the first and third quartile have a maximum length of 1.5 × interquartile range (IQR). Values < Q1 – 1.5 × IQR or > Q3 + 1.5 × IQR are considered outliers. Normal distribution was rejected for the Rpa190^GFP^ signal intensity in the case of both stationary samples according to a Kolmogorov–Smirnov test done in Origin (level set to 0.05). Hence, a two-tailed Mann–Whitney *U-*test for pairwise comparisons between groups was applied using SPSS statistics (IBM). Exact *P*-values were calculated and corrected according to Bonferroni (*P-*values were multiplied by the number of pairwise comparisons). As a non-parametric test was applied, outliers were included in the analyses (note that all *P-*values were <0.003 when excluding outliers). Bar graphs were generated in Excel (Microsoft).

## Results

To examine the interplay between Pol I transcription and Rpd3-dependent establishment of 35S rRNA gene chromatin states, we analyzed yeast cells carrying a wild-type copy of the *RPD3* gene or a complete deletion (*rpd3*Δ) upon growth to the stationary phase. To this end, changes in 35S rRNA gene chromatin states in these cells were examined during exponential growth (OD_600_ ∼0.5 (exp)), during diauxic shift (9 h after exponential growth), in the post-diauxic growth phase (48 h after exponential growth (pd)) and in the stationary phase (144 h after exponential growth (stat)) (58). To monitor 35S rDNA chromatin states, we employed psoralen photo-crosslinking as an established method (reviewed in (59)). Psoralen intercalates into DNA in open, nucleosome-depleted 35S rRNA gene chromatin, whereas nucleosomes in closed rRNA gene chromatin impair psoralen DNA incorporation (Supplementary Figure S1A, schematic representation of psoralen crosslinking analysis). Upon UV irradiation psoralen forms DNA interstrand crosslinks, resulting in heavily crosslinked 35S rRNA gene regions from open genes and sparsely crosslinked regions from closed genes. To separate these two different populations, DNA was isolated from psoralen-treated cells, cleaved with specific restriction enzymes and analyzed by native agarose gel electrophoresis and Southern blot (Figure 1B, schematic representation of fragments analyzed and probe used for hybridization). rDNA fragments derived from open 35S rRNA genes have slower mobility than the same fragments derived from closed genes. In agreement with earlier data (8,9), Rpd3-dependent closing of 35S rRNA genes determined by psoralen photo-crosslinking started during the diauxic shift in wild-type *RPD3* cells (Figure 1C, lanes 1–4, profile analyses of lanes 1 and 4 depicted in the graph below the autoradiogram, see Supplementary Table S6 for quantification). The open chromatin state was maintained in post-diauxic and stationary *rpd3*Δ cells (Figure 1C, lanes 5–8, profile analyses of lanes 5 and 8 depicted in the graph below the autoradiogram, see Supplementary Table S6 for quantification). It should be noted that already in exponentially growing cells the fraction of open 35S rRNA genes was higher in *rpd3*Δ cells when compared to *RPD3* cells (Figure 1C, lanes 1 and 5, profile analyses of lanes 1 and 5 depicted in the graph below the autoradiogram, see Supplementary Table S6 for quantification). To determine the molecular basis for these phenomena, we assessed the interaction of various protein factors with the rDNA.

### Pol I occupancy at rRNA genes is strongly reduced in the stationary phase

The establishment and maintenance of the open chromatin state depend on transcription by Pol I and binding of Hmo1 to Pol I transcribed 35S rRNA genes (38). We, therefore, investigated if the association of these factors was altered in stationary *rpd3*Δ cells when compared to *RPD3* cells. First, we analyzed alterations in Pol I association with rRNA genes upon growth to the stationary phase by ChEC analysis ((60), Supplementary Figure S1B, schematic representation of ChEC analysis). This method monitors association of proteins fused to MN with genomic loci. Specific cleavage events mediated by the respective MN fusion proteins can be detected by Southern blot analysis (Figure 1B, schematic representation of the fragments analyzed and probe used for hybridization). ChEC experiments were performed with exponentially growing *RPD3* and *rpd3*Δ cells expressing the Pol I subunit Rpa190 as MN fusion protein. Rpa190^MN^ produced multiple cuts within the 35S rRNA gene region in exponentially growing *RPD3* and *rpd3*Δ cells (Figure 2A, panels ‘exp’, lanes 1 to 10). When the cells were cultured to the stationary phase before ChEC no Rpa190^MN^-dependent cleavage events could be observed in *RPD3* cells. Weak Rpa190^MN^-dependent cleavage still occurred in *rpd3*Δ cells (Figure 2A, panels ‘stat’, lanes 1–10, see Supplementary Figure S2A and B for quantification). This result was consistent with previous observations showing that Pol I transcription was downregulated in stationary *RPD3* cells but persists at a slightly higher level in *rpd3*Δ cells (9).

In combination with psoralen photo-crosslinking, ChEC determines the preferential association of the factors with either the open or the closed 35S rRNA gene chromatin state (31,51) (Supplementary Figure S1C, schematic representation of ChEC-psoralen crosslinking analysis). In this analysis, Rpa190^MN^ specifically and fully degraded an EcoRI restriction fragment derived from psoralen accessible open 35S rRNA genes in exponentially growing *RPD3* and *rpd3*Δ cells (Figure 2B, panels ‘exp’, lanes 1–6; profile analyses of lanes 1 and 3, and 4 and 6 depicted in graphs ‘exp’ below the autoradiograms, see Supplementary Table S6 for quantification). This confirmed that Pol I is a component of open 35S rRNA gene chromatin (31,38,61). In good agreement with the *rpd3*Δ phenotype ((8,9), Figure 1C), the results of psoralen photo-crosslinking revealed significant differences in the fraction of open 35S rRNA genes in experiments with stationary *RPD3* and *rpd3*Δ cells. Whereas most of the genes were in the closed chromatin state in stationary *RPD3* cells, around half of the gene population was still in the open chromatin state in *rpd3*Δ cells (Figure 2B, panels ‘stat’, compare lane 1 with lane 4, see Supplementary Table S6 for quantification). As already suggested by ChEC analyses (Figure 2A, panels ‘stat’), no significant Rpa190^MN^ mediated degradation of psoralen crosslinked 35S rRNA gene fragments could be detected in ChEC-psoralen photo-crosslinking experiments in stationary *RPD3* cells, and only marginal degradation in stationary *rpd3*Δ cells (Figure 2B, panels ‘stat’, lanes 1–6; profile analyses of lanes 1 and 3, and 4 and 6 depicted in graphs ‘stat’ below the autoradiograms, see Supplementary Table S6 for quantification). Thus, in the stationary phase, there was no detectable Pol I association with 35S rRNA genes in *RPD3* cells, and only a very low level of Pol I associated with open 35S rRNA genes in *rpd3*Δ cells.

### Hmo1 is a component of open 35S rRNA genes in the stationary phase

Maintenance of the open 35S rRNA gene chromatin state in the absence of Pol I transcription in cell cycle-arrested cells required the HMG-box protein Hmo1 (38). In ChEC experiments with exponentially growing *RPD3* and *rpd3*Δ cells, Hmo1^MN^ mediated cleavages were observed throughout the entire Pol I transcribed 35S rRNA gene region (Figure 2A, panels ‘exp’, lanes 11–20) resembling the results obtained with Rpa190^MN^ (Figure 2A, panels ‘exp’, lanes 1–10). In contrast to Rpa190^MN^, significant Hmo1^MN^-dependent cleavage events within the 35S rRNA gene region were still observed in ChEC experiments with *RPD3* and *rpd3*Δ stationary cells (Figure 2A, panels ‘stat’, lanes 11–20). This was consistent with ChIP experiments in post-diauxic cells and stationary cells in earlier studies (8,62). Hmo1^MN^ cleavage patterns were different between exponentially growing and stationary cells. While Hmo1^MN^ cleavage events spread over the entire Pol I transcribed 35S rRNA gene region in samples from exponentially growing cells, predominant Hmo1^MN^ cleavage was observed at the 35S rDNA promoter in stationary cells (Figure 2A, compare panels ‘exp’ and ‘stat’ for lanes 13–15 and 18–20). Quantification of the cleavage events revealed that Hmo1^MN^-specific degradation of the 35S rRNA gene fragment was significantly higher in stationary *rpd3*Δ cells than in stationary *RPD3* cells (Supplementary Figure S2C and D). As expected, psoralen photo-crosslinking revealed that the fraction of open rRNA genes was higher in stationary *rpd3*Δ cells when compared with stationary *RPD3* cells (Figure 2B, panels ‘exp’ and ‘stat’, compare lane 7 with lane 10, see Supplementary Table S6 for quantification). In both exponentially growing and stationary *rpd3*Δ and *RPD3* cells, fragments derived from open rRNA genes were specifically and fully degraded by Hmo1^MN^ upon ChEC (Figure 2B, compare lanes 7–9 with lanes 10–12; profile analyses of lanes 7 and 9, and 10 and 12 are depicted in graphs ‘exp’ and ‘stat’ below the autoradiograms, see Supplementary Table S6 for quantification). These results showed that Hmo1 was a component of all open genes in exponentially growing and stationary *rpd3*Δ and *RPD3* cells. Thus, Hmo1 remained associated with 35S rRNA genes even in stationary phase when Pol I transcription was absent or at least significantly reduced.

### *RPD3* deletion affects Pol I PIC formation in the stationary phase

The above results and observations in an earlier study (9) indicated that deletion of *RPD3* maintains Pol I transcription at least to some degree in stationary cells. It is possible that persistent transcription at very low levels promotes the opening of chromatin. Concomitant recruitment of Hmo1 to transcribed rRNA genes might then support the maintenance of the open chromatin state. To determine the molecular basis of persistent Pol I transcription in the absence of Rpd3, we analyzed Pol I PIC assembly in ChIP experiments with *RPD3* and *rpd3*Δ cells expressing components of the Pol I transcription machinery fused to a TAP tag (63).

**Figure 3.**
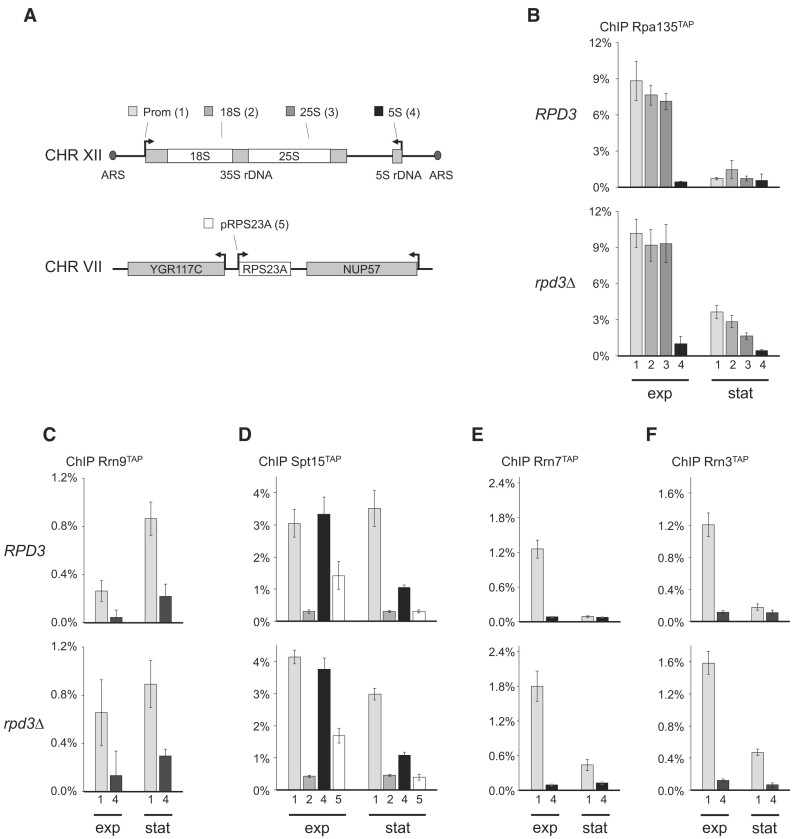
RPD3 deletion affects Pol I PIC formation in the stationary phase. *RPD3* or *rpd3*Δ strains y3078, y3116, y3118, y3120, y3122, y3124, y3126, y3136, y3327 and y3329 expressing either Rrn9^TAP^, Spt15^TAP^, Rrn7^TAP^, Rrn3^TAP^ or Rpa135^TAP^ were grown in YPAD at 30°C to exponential phase (exp) or for another 144 h to stationary phase (stat) before samples were withdrawn. Samples were subjected to ChIP experiments with IgG sepharose using the indicated bait proteins. (**A**) Schematic representation showing the locations of amplified regions within the rDNA locus (1–4) and the *RPS23A* gene (5). DNA co-precipitating with the tagged protein and from input fractions was isolated and analyzed by qPCR with different primer pairs amplifying regions 1–5 depicted in (A). (**B–F**) Efficiency of co-precipitation of the fragments with the respective TAP-fusion protein was calculated as percentages of the input DNA (% ChIP) and are depicted in bar graphs. The mean and standard deviation were derived from three independent ChIP experiments analyzed each in triplicate qPCR reactions (*n* = 9). Representative results of two biological replicates are shown.

A fusion protein of the second largest Pol I subunit, Rpa135^TAP^, efficiently co-precipitated DNA fragments spanning the entire Pol I transcribed 35S rRNA gene region in exponentially growing *RPD3* and *rpd3*Δ cells, but not a DNA fragment encompassing the Pol III transcribed 5S rRNA gene including the promoter region (Figure 3B, compare fragments 1–3 with fragment 4 in ‘exp’ samples, see Figure 3A for the location of the rDNA regions amplified by qPCR). In stationary *RPD3* cells, co-precipitation of 35S rRNA gene fragments with Rpa135^TAP^ was reduced to background levels, indicating that Pol I association with rRNA genes was strongly impaired (Figure 3B, compare fragments 1–3 with fragment 4 in ‘*RPD3*’ ‘stat’ samples). In stationary *rpd3*Δ cells, reduced but significant co-precipitation of DNA fragments of the Pol I transcribed rRNA gene region was observed (Figure 3B, compare fragments 1–3 with fragment 4 in ‘*rpd3*Δ’ ‘stat’ samples). These results were consistent with ChEC analyses (Figure 2A; Supplementary Figure S2A and B) suggesting that Pol I transcription was generally downregulated in stationary *RPD3* and *rpd3*Δ cells while residual transcription persisted in the absence of Rpd3.

ChIP experiments with a TAP-fusion protein of the UAF component Rrn9 led to co-precipitation of a 35S rDNA promoter fragment in exponentially growing and stationary *RPD3* and *rpd3*Δ cells compared to a DNA-fragment encompassing the Pol III transcribed 5S rRNA gene (Figure 3C, compare fragments 1–4). This indicated that UAF associated with 35S rDNA promoters in exponentially growing and stationary yeast cells, which was in good accordance with the results of an earlier study (40).

ChIP experiments with TAP-tagged yeast TBP, Spt15^TAP^, did not show differences in co-precipitation of 35S rDNA promoter fragments from exponentially growing or stationary *RPD3* and *rpd3*Δ cells (Figure 3D, fragment 1). As expected for a promoter-bound PIC component, no significant co-precipitation of a fragment within the Pol I transcribed 18S rRNA coding region with Spt15^TAP^ was observed (Figure 3D, fragment 2). In contrast to ChIP with Rrn9^TAP^, a DNA fragment encompassing the Pol III transcribed 5S rRNA gene was enriched to the same extent as the 35S rDNA promoter fragment from exponentially growing *RPD3* and *rpd3*Δ cells in ChIP experiments with Spt15^TAP^ (Figure 3D, compare fragment 1 and 4 in ‘exp’ samples). This is because TBP is a PIC component for all three nuclear RNA Pols and associates robustly with the 5S rRNA gene promoter, which was included in fragment 4 (64,65). Accordingly, slightly less—but robust—co-precipitation with Spt15^TAP^ was also observed for a fragment containing the Pol II dependent promoter of the *RPS23A* gene coding for a ribosomal protein of the small subunit from exponentially growing *RPD3* and *rpd3*Δ cells (Figure 3D, fragment 5). Contrary to the 35S rDNA promoter fragment, the amounts of 5S rDNA and *RPS23A* promoter fragments co-precipitating with Spt15^TAP^ in stationary *RPD3* and *rpd3*Δ cells were strongly reduced and at background levels, respectively (Figure 3D, compare co-precipitation of fragments 4, 5 with fragment 1 in ‘exp’ and ‘stat’ samples). These results suggested that the association of TBP with Pol I, II and III-dependent promoters was differentially regulated upon growth to the stationary phase. Furthermore, there were no significant differences in TBP association with the investigated promoters between *RPD3* or *rpd3*Δ cells, indicating that Rpd3 did not regulate this step of PIC formation at the respective loci.

Robust co-precipitation of a 35S rDNA promoter fragment with the CF component Rrn7^TAP^ was observed in exponentially growing *RPD3* and *rpd3*Δ cells. The co-precipitation was reduced to background levels in stationary *RPD3* cells (Figure 3E, compare the co-precipitation of fragment 1 with fragment 4 in ‘exp’ and ‘stat’ samples). In contrast, there was lower but still significant co-precipitation of this fragment in stationary *rpd3*Δ cells (Figure 3E, compare the co-precipitation of fragment 1 in ‘*rpd3*Δ’ ‘exp’ and ‘*rpd3*Δ’ ‘stat’ samples). These results correlated well with ChIP experiments using Rrn3^TAP^ and Rpa135^TAP^ expressing cells (Figure 3B and F compare co-precipitation of fragments 1–4 in ‘stat’ samples). This suggested reduced but significant association of CF, Rrn3 and Pol I with the 35S rDNA promoter in stationary *rpd3*Δ cells, whereas the association of these proteins was almost at background levels in stationary *RPD3* cells.

ChEC experiments with strains expressing MN fusion proteins of the UAF component Rrn9, yeast TBP Spt15 and the CF component Rrn7 were in good agreement with the results from the above ChIP experiments (Supplementary Figure S3). Whereas Rrn9^MN^ and Spt15^MN^ showed very similar 35S rDNA promoter-specific cleavage events in exponentially growing and stationary *RPD3* and *rpd3*Δ cells (Supplementary Figure S3A and B), Rrn7^MN^-mediated cleavage was at background levels in stationary *RPD3* cells while significant cleavage was detected in stationary *rpd3*Δ cells (Supplementary Figure S3C). Taken together these data suggest that the deletion of *RPD3* de-regulated Pol I PIC assembly at the level of CF (and subsequently Rrn3/Pol I) recruitment to the 35S rDNA promoter.

### *RPD3* deletion increases nucleolar levels of CF subunits in stationary cells

Deregulation of Pol I transcription in stationary *rpd3*Δ cells was also investigated by analyzing nucleolar localization of components of the Pol I transcription machinery using live cell imaging. To this end, *RPD3* and *rpd3*Δ yeast strains were generated expressing various fusion proteins from their endogenous genomic location with a C-terminal GFP and co-expressing endogenously triple mCherry (3mCherry) tagged Hmo1. Hmo1 is an acknowledged nucleolar marker (66) and remains associated with the rDNA independent of the growth phase (8,62) (Figure 2). Exponentially growing and stationary cells were subjected to live cell fluorescence microscopy.

**Figure 4.**
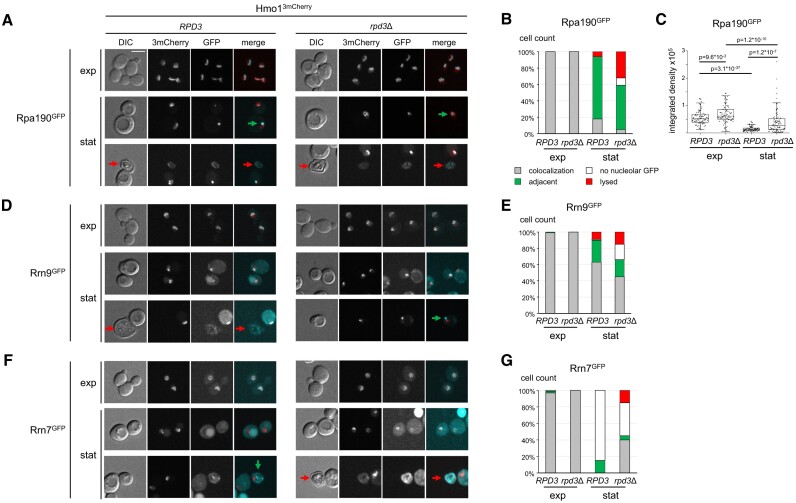
*RPD3*deletion increases nucleolar levels of CF subunits in stationary cells. *RPD3* or *rpd3*Δ strains, W17017, W17018, W17021, W17022, W17341 and W17339, expressing Hmo1^3mCherry^ and either Rpa190^GFP^, Rrn9^GFP^ or Rrn7^GFP^ were grown in YPAD+ at 30°C to exponential phase or for another 129 h to stationary phase before samples were withdrawn and subjected to live-cell fluorescence microscopy. (**A**, **D**, **F**) Micrographs after DIC imaging and fluorescence imaging of 3mCherry or GFP are depicted. In merge images, GFP is depicted in cyan and 3mCherry in red. Red and green arrows point to lysed cells and cells classified to have ‘adjacent’ fluorescent signals, respectively (see below). (**B**, **E**, **G**) A population of 100 cells was classified into four different cellular phenotypes according to the observed fluorescence as ‘adjacent’ signals, ‘colocalization’ of GFP and 3mCherry, ‘no nucleolar GFP,’ or judged by the appearance in DIC images as ‘lysed.’ The cell count for each phenotype is depicted in bar graphs as the percentage of the total population. Color coding of the bar graphs is depicted in (B). (**C**) The box plot shows the overall integrated density of the Rpa190^GFP^ signal within the thresholded signal of Hmo1^3mCherry^ for 90 cells for the indicated yeast strain. Statistical analyses are described in the ‘Materials and Methods’ section. Representative results of two biological replicates are shown.

Rpa190^GFP^ co-localized with Hmo1^3mCherry^ in exponentially growing *RPD3* and *rpd3*Δ cells (Figure 4A, panels ‘exp’). In the stationary phase, however, no strict overlap of the GFP and 3mCherry fluorescence was observed in most *RPD3* and *rpd3*Δ cells. Instead, the Rpa190^GFP^ signal appeared as a focused dot just adjacent to the broader Hmo1^3mCherry^ signal (Figure 4A, panels ‘stat’, dot-like GFP signals are labeled with green arrows; see Figure 4B for the percentage for the observed cellular phenotypes within a fixed number of cells). Notably, in stationary cells, a population of lysed cells identified by their characteristic appearance in DIC imaging was observed. Cell lysis was significantly increased in *rpd3*Δ cells (Figure 4A, lysed cells are labeled with red arrows) consistent with earlier observations (67). Supplementary Figure S4A and B shows the results of experiments with untagged *RPD3* and *rpd3*Δ strains to illustrate the phenomenon. In these experiments, it could be observed that lysed cells often exhibited autofluorescence (68). Further quantitative image analysis revealed that the overall Rpa190^GFP^ signal within a region defined by a threshold of Hmo1^3mCherry^ fluorescence (integrated density) strongly decreased in stationary *RPD3* cells when compared with the integrated density in exponentially growing cells (Figure 4C). Instead, the Rpa190^GFP^ signal was partially maintained in *rpd3*Δ cells (Figure 4C). This correlated well with the observed increase in Pol I association with 35S rRNA genes in stationary *rpd3*Δ cells when compared to stationary *RPD3* cells in ChEC and ChIP experiments (Figures 2 and 3B) further supporting that Pol I transcription partially persisted in stationary *rpd3*Δ cells.

A GFP fusion protein of the UAF component Rrn9, Rrn9^GFP^, co-localized with Hmo1^3mCherry^ in exponentially growing and stationary *RPD3* and *rpd3*Δ cells (Figure 4D, panels ‘exp’ and ‘stat’, see Figure 4E for quantification of cellular phenotypes). As observed for Rpa190^GFP^ in stationary *RPD3* and *rpd3*Δ cells Rrn9^GFP^ fluorescence either overlapped with Hmo1^3mCherry^ fluorescence or appeared adjacent to the Hmo1^3mCherry^ signal. There was also a small subpopulation of cells with Hmo1^3mCherry^ signal but no detectable GFP fluorescence. Very similar observations were made for cells expressing GFP fusion proteins of the UAF components Rrn5 and Uaf30 (Supplementary Figure S4C–F). These observations showed that UAF co-localized well with the rDNA-binding protein Hmo1 in stationary *RPD3* and *rpd3*Δ cells.

As observed for Pol I and UAF, a GFP fusion protein of the CF component Rrn7, Rrn7^GFP^, co-localized with Hmo1^3mCherry^ in exponentially growing *RPD3* and *rpd3*Δ cells (Figure 4F, panels ‘exp’, see Figure 4G for quantification of cellular phenotypes). The large majority of stationary *RPD3* cells did not reveal a detectable GFP signal despite a clear Hmo1^3mCherry^ fluorescence (Figure 4F, panels ‘stat’, see Figure 4G for quantification of cellular phenotypes). In contrast, significant Rrn7^GFP^ did still co-localize with Hmo1^3mCherry^ in more than one-third of the analyzed stationary *rpd3*Δ cells (Figure 4F and G, ‘stat’). In a significant fraction of the stationary *RPD3* cells, Rrn7^GFP^-mediated fluorescence was observed adjacent to the Hmo1^3mCherry^ signal (Figure 4F, panel ‘*RPD3*’ ‘stat’, adjacent fluorescence labeled by a green arrow). Very similar results were obtained for a strain expressing a GFP fusion protein of the CF component Rrn6 (Supplementary Figure S4G and H). This indicates that the deletion of *RPD3* led to the maintenance of significant amounts of nucleolar CF in stationary cells, whereas in the presence of Rpd3, no significant nucleolar CF levels could be detected.

Taken together, live cell imaging supports the observations of ChIP and ChEC experiments (Figure 3 and Supplementary Figure S3) indicating that the lack of (nucleolar) CF prevented complete Pol I PIC formation in *RPD3* cells. In turn, deletion of *RPD3* led to persistent levels of nucleolar CF which was available for binding to 35S rDNA promoters allowing Pol I transcription and maintenance of open rRNA genes.

### *RPD3* deletion increases protein levels of UAF and CF in stationary cells

A simple explanation for the increased CF nucleolar localization and association with the 35S rDNA promoter in stationary *rpd3*Δ cells when compared to stationary *RPD3* cells could be that the cellular protein amounts of CF components are different in the presence or absence of Rpd3. In this regard, a recent genome-wide analysis in *RPD3* and *rpd3*Δ cells analyzed steady state transcript levels upon growth to the stationary phase (67). It was reported that the general strong downregulation of transcripts of most yeast genes in stationary *RPD3* cells was attenuated in stationary *rpd3*Δ cells. The published dataset revealed that transcripts coding for the factors analyzed in the present study were present in 6- to 37-fold excess in stationary *rpd3*Δ cells compared to stationary *RPD3* cells ((67) accession GSE67149).

**Figure 5.**
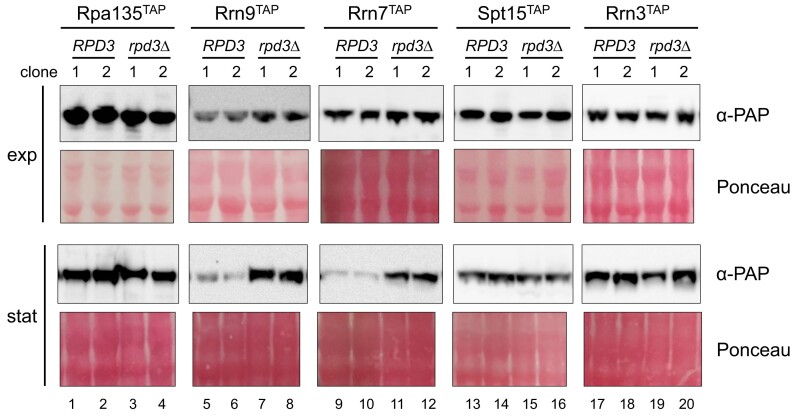
*RPD3*deletion increases protein levels of UAF and CF components in stationary cells. *RPD3* or *rpd3*Δ strains y3078, y3079, y3116, y3117, y3118, y3119, y3120, y3121, y3122, y3123, y3124, y3125, y3126, y3127, y3136, y3137, y3327, y3328, y3329 and y3330 expressing either Rrn9^TAP^, Spt15^TAP^, Rrn7^TAP^, Rrn3^TAP^ or Rpa135^TAP^ were grown in YPAD at 30°C to exponential phase or for another 144 h to stationary phase before samples were withdrawn. Whole-cell extracts were prepared from two independent clones for all strains (1,2) and analyzed in a western blot. After transfer to the membrane proteins were visualized by staining with Ponceau S (Ponceau). TAP-fusion proteins were detected with peroxidase antiperoxidase soluble complex (α-PAP). Images visualizing chemiluminescence signals resulting from the peroxidase activity are shown. Whole-cell extracts from the same number of cells were loaded for each sample set derived from strains expressing a certain TAP-fusion protein. The whole-cell extracts were adjusted to contain proteins from 2.25 × 10^6^ to 9 × 10^6^ cells for samples taken during exponential growth, and 1.8 × 10^7^ cells for samples taken in the stationary phase. Images of chemiluminescence signals on the full membranes as well as Ponceau S staining of the full membranes before incubation with the antibodies are shown in Supplementary Figure S5A. Representative results of two biological replicates are shown.

In the latter study, it was not investigated if the differences in the transcriptomes correlated with alterations in protein amounts. To this end, whole-cell protein extracts from strains expressing the TAP-fusion proteins analyzed in the above ChIP analyses (Figure 3) were prepared from exponentially growing and stationary cells. For each *RPD3* and *rpd3*Δ pair, two independent clones were analyzed. Proteins were extracted from the same number of cells, separated by SDS-PAGE, and subjected to western blot analysis. For less abundant TAP-fusion proteins and samples from stationary cells, more extract was loaded to allow better detection. Blots were stained with Ponceau S to ensure equal protein loading in the lanes with extracts containing the respective TAP-fusion proteins (Figure 5, panels ‘Ponceau,’ see Supplementary Figure S5 for images of the full membrane). The TAP-fusion proteins were detected by a peroxidase–antiperoxidase immunocomplex (α-PAP) (63). The levels of a particular TAP-fusion protein were similar in exponentially growing *RPD3* and *rpd3*Δ cells (Figure 5, panel ‘exp’ ‘α-PAP,’ see Supplementary Figure S5 for images of the full membranes). One exception was Rrn9^TAP^ whose levels were slightly increased in extracts of *rpd3*Δ cells. Rpa135^TAP^, Spt15^TAP^ and Rrn3^TAP^ levels were also similar in stationary *RPD3* and *rpd3*Δ cells (Figure 5, panel ‘stat’ ‘α-PAP,’ lanes 1–4, lanes 13–16 and 17–20, see Supplementary Figure S5 for images of the full membranes). However, levels of Rrn9^TAP^ and Rrn7^TAP^ were significantly lower in *RPD3* cells when compared to the levels in *rpd3*Δ cells (Figure 5, panel ‘stat’ ‘α-PAP,’ lanes 5–8, and lanes 9–12, see Supplementary Figure S5 for images of the full membranes). Thought as a loading control, the western blot membrane was subsequently hybridized with a rat monoclonal antibody directed against yeast Tub1 (Supplementary Figure S5, panel ‘α-tubulin’). Remarkably, also Tub1 levels were increased in *rpd3*Δ cells. These results showed that protein levels of Rrn9 and Rrn7 in the stationary phase were increased in *rpd3*Δ cells compared to *RPD3* cells. In good agreement with these results, western blot experiments analyzing the cellular amounts of GFP fusion proteins investigated in Figure 4 indicated increased protein levels of Uaf30^GFP^, Rrn9^GFP^ and Rrn7^GFP^ but not for Rrn5^GFP^ and Rpa190^GFP^ in stationary *rpd3*Δ cells compared to *RPD3* cells (Supplementary Figure S5B, lanes 7, 8, 15, 16, 23, 24, 31, 32, 47 and 48). In strains expressing Rrn6^GFP^ the GFP fusion protein could not be detected in stationary *RPD3* cells but at very low levels in *rpd3*Δ cells, indicating increased levels for a second CF component in the absence of Rpd3 (Supplementary Figure S5B, lanes 39 and 40). Increased CF protein levels in stationary *rpd3*Δ cells could explain the increased recruitment of CF to the 35S rDNA promoter, whereas increased protein levels of Uaf30 and Rrn9 did not significantly increase Rrn9 interaction with the 35S rDNA promoter (Figure 3 and Supplementary Figure S3). The analysis also revealed that the globally increased transcript levels in stationary *rpd3*Δ cells (67) were not necessarily correlated with increased protein levels.

### Residual RNA Pol I transcription maintains the open chromatin state in stationary *rpd3Δ* cells

**Figure 6.**
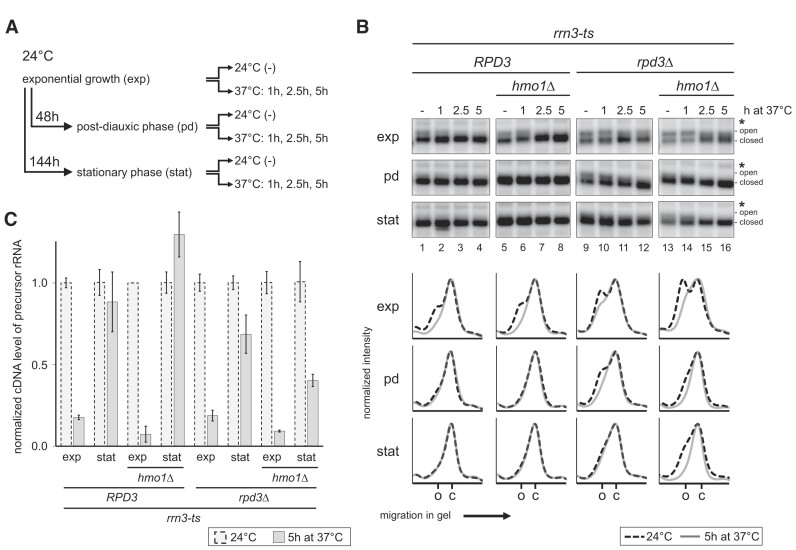
Residual RNA Pol I transcription maintains the open chromatin state in stationary *rpd3*Δ cells. (**A**) Outline of the experiment. *RPD3* or *rpd3*Δ strains NOY1075, y2921, y2945 and y2947, carrying an *HMO1* wild-type allele or a complete deletion of the gene (*hmo1*Δ) and expressing a temperature-sensitive mutant of Rrn3 (*rrn3-ts*) were grown in YPAD at 24°C to exponential phase (exp), for another 48 h to post-diauxic phase (pd), and for 144 h to stationary phase (stat). At each time point samples were withdrawn (0 h), a fraction of the respective culture was shifted to 37°C, and samples were withdrawn at the indicated times. (**B**) All samples were subjected to psoralen photo-crosslinking and analyzed as described in the legend of Figure 1C. Autoradiograms show results for the 2.9 kb 25S rDNA fragment. The positions of psoralen crosslinked fragments derived from 'open' rRNA genes and from ‘closed’ rRNA genes are indicated on the right. The position of a band resulting from incomplete digestion of psoralen-crosslinked DNA is labeled by an asterisk. Graphs at the bottom show analyses of the radioactivity profile in the lanes with samples of cells cultured at 24°C (0 h) (dotted black lines) and of cells after 5 h incubation at 37°C (gray lines). The positions of the peaks corresponding to the fragments derived from open (o) and closed (c) rRNA genes are labeled on the *x*-axis of the graphs at the bottom. (**C**) RNA was isolated from the above strains at exponential and stationary phase at 24°C, and after 5 h at 37°C respectively. The RNA was subjected to RT-qPCR analysis using primers flanking the A3 processing site detecting cDNA of an unstable 35S rRNA precursor and primers detecting cDNA of 5S rRNA. The amount of the unstable 35S rRNA precursor relative to 5S rRNA was determined for each sample. The bar graph depicts the relative 35S rRNA amount after 5 h at 37°C normalized to the relative 35S rRNA amount at 24°C for each strain. The mean and standard deviation were derived from two independent biological replicates analyzed each in duplicate or triplicate qPCR reactions. Representative results of three (Figure 6B) and two (Figure 6C) biological replicates are shown.

Taken together, the results suggested that increased CF protein levels in stationary *rpd3*Δ cells led to PIC formation at multiple 35S rDNA promoters supporting residual Pol I transcription, Hmo1 recruitment, and, thus, establishment and maintenance of open 35S rRNA gene chromatin. To test this hypothesis, *RPD3* and/or *HMO1* were deleted in a strain expressing a temperature-sensitive allele of the essential Pol I transcription factor Rrn3 (*rrn3-ts*). In this background Pol I transcription could be conditionally inactivated by shifting the growth temperature from 24°C to 37°C and further incubation for 2.5 h (40). *RPD3* and *rpd3*Δ cells carrying either a wild-type allele or a complete deletion of *HMO1* were grown to exponential phase at 24°C and samples were withdrawn. A fraction of the cells was then incubated at 37°C from which further samples were withdrawn after various times. This procedure was repeated with the remainder of the culture grown to the post-diauxic phase, and to the stationary phase at 24°C for 48 and 144 h, respectively (Figure 6A shows a flow diagram of the experimental design). Each of the samples was subjected to psoralen photo-crosslinking analysis. In exponentially growing *rrn3-ts RPD3* and *rrn3-ts RPD3 hmo1*Δ cells, shut-down of Pol I transcription led to the closing of the open 35S rRNA gene population in the presence and absence of Hmo1 (Figure 6B, panels ‘exp’, lanes 1–8; profile analyses of lanes 1 and 4, and 5 and 8 are depicted in graphs ‘exp’ below the autoradiograms, see Supplementary Table S6 for quantification). This was in good agreement with earlier results showing that Pol I transcription is required to maintain the open rRNA gene chromatin state in replicating cells (38). When these cells were grown to the post-diauxic or the stationary phase at the permissive temperature, most of the rRNA genes adapted to the closed chromatin state (Figure 6B, panels ‘pd’ and ‘stat’, lanes 1 and 5, see Supplementary Table S6 for quantification). Shifting these cells to the restrictive temperature did not lead to detectable changes in psoralen accessibility of the closed rRNA genes (Figure 6B, panels ‘pd’ and ‘stat’, lanes 1–8; profile analyses of lanes 1 and 4, and 5 and 8 are depicted in graphs ‘pd’ and ‘stat’ below the autoradiograms, see Supplementary Table S6 for quantification). Exponentially growing *rrn3-ts rpd3*Δ or *rrn3-ts rpd3*Δ *hmo1*Δ cells showed a higher fraction of open rRNA genes than the corresponding *RPD3* cells and a gradual but incomplete closing of 35S rRNA gene chromatin upon temperature shift to 37°C for 5 h (Figure 6B, panels ‘exp’, lanes 9–16; profile analyses of lanes 9 and 12, and 13 and 16 are depicted in graphs ‘exp’ below the autoradiograms, see Supplementary Table S6 for quantification). Thus, Pol I transcription was also required to maintain an open rRNA gene chromatin state in these genetic backgrounds, although the kinetics of 35S rRNA gene chromatin closing appeared to be delayed. In post-diauxic and stationary *rrn3-ts rpd3*Δ and *rrn3-ts rpd3*Δ *hmo1*Δ cells, a significant fraction of rRNA genes was still in the open chromatin state (Figure 6B, panels ‘pd’ and ‘stat’, lanes 9 and 13, see Supplementary Table S6 for quantification). It should be noted that the separation of DNA fragments derived from open and closed rRNA genes in post-diauxic and stationary cells was not as good as it was in exponentially growing cells. Importantly, upon shift to the restrictive temperature, all rRNA genes in post-diauxic and stationary *rrn3-ts rpd3*Δ *hmo1*Δ cells adapted the closed chromatin state (Figure 6B, panels ‘pd’ and ‘stat’, lanes 13–16; profile analyses of lanes 13 and 16 depicted in graphs ‘pd’ and ‘stat’ below the autoradiograms, see Supplementary Table S6 for quantification). However, only incomplete closing of rRNA gene chromatin was observed in post-diauxic and stationary *rrn3-ts rpd3*Δ cells in the presence of Hmo1 (Figure 6B, panels ‘pd’ and ‘stat’, lanes 9–12; profile analyses of lanes 9 and 12 depicted in graphs ‘pd’ and ‘stat’ below the autoradiograms). In control experiments with the corresponding wild-type *RRN3* strains, temperature shift to 37°C for up to 5h did not significantly influence psoralen accessibility of 35S rRNA genes (Supplementary Figure S6 and Supplementary Table S6 for quantification). To verify that the observed closing of rRNA gene chromatin in *rrn3-ts* strains upon temperature shift went along with downregulation of rRNA gene transcription by Pol I, rRNA precursor levels were measured by RT-qPCR. In all *rrn3-ts* strains temperature shift resulted in a significant downregulation of rRNA precursor in exponentially growing cells (Figure 6C). In stationary cells temperature shift led only in *rrn3-ts rpd3*Δ and *rrn3-ts rpd3*Δ *hmo1*Δ cells to decreased rRNA precursor levels indicating that in these strains temperature shift resulted in downregulation of persistent rRNA gene transcription. These data indicated that residual Pol I transcription was required to maintain open 35S rDNA chromatin in post-diauxic and stationary *rpd3*Δ cells, and that open rRNA genes might be stabilized by Hmo1 in the absence of residual Pol I transcription.

### CF is a limiting factor for the establishment of open rRNA gene chromatin

**Figure 7.**
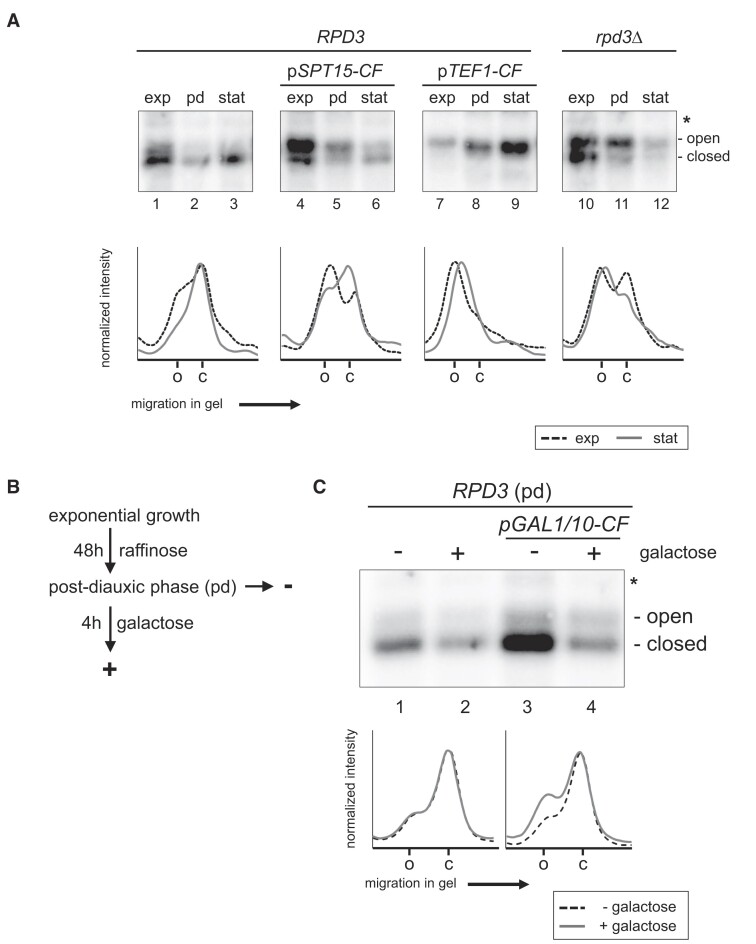
CF is a limiting factor for the establishment of open rRNA gene chromatin. (**A**) *RPD3* strains NOY505, y4966 and y4969 expressing CF genes from the endogenous locus and carrying a genomically integrated expression cassette for all three CF proteins under the control of either the p*SPT15* or p*TEF1* promoter (p*SPT15-CF*, p*TEF1-CF*), were grown in YPAD at 30°C. They were cultured until reaching exponential phase (exp), followed by an additional 48 h to reach post-diauxic phase (pd), and then grown for 144 h to stationary phase (stat). The *rpd3*Δ strain y2919 was cultured in parallel as a control. Samples were withdrawn at the time points indicated in the figure and subjected to psoralen photo-crosslinking and analyzed as described in the legend of Figure 1C. Autoradiograms show results for the 2.9 kb 25S rDNA fragment. The positions of psoralen crosslinked fragments derived from 'open' rRNA genes and from ‘closed’ rRNA genes are indicated on the right. The position of a band resulting from incomplete digestion of psoralen-crosslinked DNA is labeled by an asterisk. Graphs on the bottom show analyses of the radioactivity profile in the lanes with samples of cells in exponential phase (dotted black lines) and of cells in stationary phase (gray lines). The positions of the peaks corresponding to the fragments derived from open (o) and closed (c) rRNA genes are labeled on the *x*-axis of the graphs at the bottom. (**B**) Outline of the experiment analyzed in (**C**). (**C**) *RPD3* strains NOY505 and y4970 expressing CF genes from the endogenous locus and carrying a genomically integrated expression cassette for all three CF proteins under the control of a p*GAL1/10* promoter (p*GAL1/10-CF*) were grown in 2xYPAR for 48 h to post-diauxic shift phase (pd). Galactose was added to a final concentration of 2% (w/v) and incubation at 30°C was continued for 4 h. Samples were withdrawn before (-) and after galactose addition (+) and subjected to psoralen photo-crosslinking and analyzed as described in the legend of Figure 1C. Autoradiograms show results for the 2.9 kb 25S rDNA fragment. The positions of psoralen crosslinked fragments derived from 'open' rRNA genes and from ‘closed’ rRNA genes are indicated on the right. The position of a band resulting from incomplete digestion of psoralen-crosslinked DNA is labeled by an asterisk. Graphs at the bottom show analyses of the radioactivity profile in the lanes with samples of cells before (dotted black lines) and after (gray lines) the addition of galactose. The positions of the peaks corresponding to the fragments derived from open (o) and closed (c) rRNA genes are labeled on the *x*-axis of the graphs at the bottom. Representative results of four (Figure 7A) and three (Figure 7C) biological replicates are shown.

Together, the above data suggest that increased CF amounts and in turn residual Pol I transcription can prevent the closing of rRNA gene chromatin in stationary *rpd3*Δ cells. This could indicate that CF is a limiting factor for the establishment of open rRNA gene chromatin. To test this possibility, we established *RPD3* wild-type strains carrying genomically integrated expression cassettes for all three CF proteins either under the control of the moderate p*SPT15* promoter (p*SPT15-CF*) or the strong p*TEF1* promoter (p*TEF1-CF*). RT-qPCR analysis indicated that RNA levels of the overexpressed genes in exponentially growing cells were increased up to 5-fold in p*SPT15-CF* strains and up to 23-fold in p*TEF1-CF* strains (Supplementary Figure S7). These yeast strains were cultured to exponential, the post-diauxic and the stationary phase and subjected to psoralen photo-crosslinking analyses. In p*SPT15-CF* strains a strong increase in the fraction of open rRNA genes compared to the isogenic wild-type strain was observed in exponentially growing cells (Figure 7A, compare lanes 1 and 4, profile analyses of lanes 1 and 4 are depicted in graphs below the autoradiograms, see Supplementary Table S6 for quantification). Open rRNA gene chromatin was partly maintained in p*SPT15-CF* cells in the stationary phase, although the fraction of open rRNA genes was reduced when compared to exponentially growing cells (Figure 7A, compare lanes 4 and 6, profile analyses of lanes 4 and 6 are depicted in graph below the autoradiogram, see Supplementary Table S6 for quantification). In the p*TEF1-CF* strain the fraction of open rRNA genes was even more increased such that the large majority of rRNA genes was in the open chromatin state even in the stationary phase (Figure 7A, lanes 7–9, profile analyses of lanes 7 and 9 are depicted in graph below the autoradiogram, see Supplementary Table S6 for quantification). The fraction of open rRNA genes in the stationary p*TEF1-CF* strain even surpassed the fraction of open rRNA genes in stationary *rpd3*Δ cells (Figure 7A, compare lanes 9 and 12, profile analyses of lanes 9 and 12 are depicted in graphs below the autoradiograms, see Supplementary Table S6 for quantification). It should be noted that the psoralen incorporation in open rRNA gene chromatin decreased in post-diauxic and stationary cells, as indicated by a downshift of the band derived from open rRNA genes in the Southern blot analyses (Figure 7A, for example lanes 7–9, profile analyses of lanes 7 and 9 are depicted in graph below the autoradiogram). This suggests that increased Pol I occupancy at rRNA genes may enhance psoralen accessibility of the DNA template. Thus, a reduction in Pol I occupancy at rRNA genes would lead to a decrease in psoralen incorporation. In support to this hypothesis, a similar downshift of the band derived from open rRNA genes was observed in cell cycle arrested cells upon inactivation of Pol I transcription (Figure 6C in (38)), or in cells with varying amounts of rRNA gene copies (Supplementary Figure S4 in (69)).

Additionally, a strain for conditional expression of CF proteins under the control of the galactose inducible bidirectional p*GAL1/10* promoter was established (p*GAL1/10-CF*). These cells were grown in raffinose containing media to post-diauxic phase and then incubated for 4 h in the presence of galactose (Figure 7B). Cells before and after addition of galactose were subjected to psoralen photo-crosslinking analyses. In the presence of galactose opening of rRNA genes was observed in p*GAL1/10-CF* but not in the isogenic wild-type strain (Figure 7C, compare lanes 2 and 4, profile analyses of lanes 1 and 2, and 3 and 4 are depicted in graphs below the autoradiograms, see Supplementary Table S6 for quantification).

Taken together, overexpression of CF components in *RPD3* wild-type cells is sufficient to mimic the open rRNA gene chromatin phenotype observed in stationary *rpd3Δ* strains. The results further indicate that the abundance of CF proteins limits the number of open rRNA genes during exponential growth and in the stationary phase.

## Discussion

In this study, we provide closer insights into the interplay between Pol I transcription and rRNA gene chromatin structure upon growth to the stationary phase. Previous data suggested that downregulation of Pol I transcription upon growth to the stationary phase involved two independent pathways, TOR-signaling dependent reduction of transcription initiation and Rpd3-dependent chromatin alterations limiting the number of open 35S rRNA genes (9,42). Here we show that in the absence of Rpd3 increased protein levels of CF components correlate with increased PIC formation at 35S rDNA promoters and low levels of Pol I transcription in stationary yeast cells. Persistent Pol I transcription then maintains the open 35S rRNA gene chromatin state in the stationary phase. The open chromatin state might further be stabilized by the HMG-box protein Hmo1. Thus, the increased fraction of open rRNA genes observed in stationary *rpd3*Δ cells are likely the consequence of an increased number of initiation-competent 35S rDNA promoters and residual Pol I transcription. In support of this hypothesis increased protein levels of CF components are sufficient to maintain the open rRNA gene chromatin state in the stationary phase even in the presence of Rpd3.

### The roles of Pol I transcription, Hmo1 and Rpd3 in the establishment and maintenance of rRNA gene chromatin states

The HMG-box protein Hmo1 was shown to maintain open rRNA gene chromatin in the absence of Pol I transcription (38). Thus, it was a prime candidate for a factor stabilizing the open rRNA gene chromatin state in stationary *rpd3*Δ cells. Consistent with this hypothesis, Hmo1 was still associated with rDNA in post-diauxic and stationary *rpd3*Δ cells ((8), this study, Figure 2 and Supplementary Figure S2C and D). However, the deletion of *HMO1* in *rpd3*Δ cells could not suppress the open 35S rRNA gene phenotype in the stationary phase (Figure 6B and Supplementary Figure S6). Only when the essential Pol I transcription factor Rrn3 was conditionally inactivated in stationary *hmo1*Δ *rpd3*Δ cells, the complete closing of 35S rRNA genes could be observed (Figure 6B). This indicated that (i) residual Rrn3-Pol I complex might still be initiation competent in stationary *rpd3*Δ cells and (ii) that Pol I transcription of rRNA genes, albeit at a very low level, was required to maintain the open chromatin state in stationary *hmo1*Δ *rpd3*Δ cells. Although Hmo1 was not required to maintain open rRNA genes in stationary *rpd3*Δ cells, there was evidence that it stabilized open rRNA gene chromatin upon conditional inactivation of Pol I transcription in stationary *rpd3*Δ cells (Figure 6B). This was similar to observations in a previous study, in which Hmo1 was required to maintain open 35S rRNA genes in exponentially growing *RPD3* wild-type cells after cell cycle arrest and shut-down of Pol I transcription (38). In stationary and cell cycle-arrested cells the genomic DNA does not undergo replication, which is likely the dominant process to revert open 35S rRNA genes to the closed chromatin state (38). Thus, in cell cycle arrested and stationary cells Hmo1 bound to 35S rRNA genes might prevent replication-independent nucleosome deposition.

Results of recent *in vitro* experiments have shown that Hmo1 may destabilize nucleosomes (70). This led to the hypothesis that Hmo1 recruitment may stimulate nucleosome disassembly to establish open 35S rRNA gene chromatin. The results presented here and in an earlier study (38) indicate that Hmo1 is not required for the establishment of the open chromatin state (Figure 6B and Supplementary Figure S6). It cannot be excluded, however, that the presence of Hmo1 may support chromatin opening.

It was previously suggested, that Rpd3 acts as a chromatin stabilization module needed for the assembly or maintenance of nucleosomes at 35S rRNA genes in stationary cells (8). However, the data presented here indicate that nucleosomes can be stably assembled at 35S rRNA genes in stationary *rpd3*Δ cells upon inhibition of Pol I transcription (Figure 6B). This suggests that neither the chromatin stabilizing effect of Rpd3 nor its lysine-deacetylase activity is required to establish closed 35S rRNA gene chromatin. However, it cannot be excluded that these functions of Rpd3 may support the latter process.

### The roles of TOR inhibition and Rpd3 in the regulation of Pol I transcription upon growth to stationary phase

To date, the roles of Rpd3 and TOR in regulating Pol I transcription in different growth phases have been controversially discussed in the literature. One study concluded that Rpd3-dependent nucleolar (chromatin) compaction might be the cause for impaired Pol I transcription upon inactivation of TOR signaling (71). Opposed to this model that Rpd3 is a downstream target of TOR, other studies argued that TOR and Rpd3 independently regulate Pol I transcription and rRNA gene chromatin structure, respectively (9,42). Thus, it was shown that 35S rRNA production was efficiently downregulated upon TOR inactivation by rapamycin and upon growth to stationary phase in both *RPD3* and *rpd3*Δ cells.

Downregulation of Pol I transcription upon growth to the stationary phase might be primarily due to a reduction in the level of the Rrn3-Pol I complex (26). Similarly, Rrn3-Pol I complex stability or cellular levels were also affected by the inactivation of TOR signaling upon rapamycin treatment or amino acid depletion (40,72). The reduction in the cellular Rrn3-Pol I complex level was suggested to be the result of proteasome-dependent degradation of Rrn3 in the context of a TOR-dependent general inhibition of translation (72). The decrease in Rrn3-Pol I complex upon rapamycin treatment correlated with a drastic reduction in RNA synthesis (40,72). Nevertheless, a regulated reduction of Rrn3-Pol I complex did not phenocopy rapamycin treatment with regard to the complete inhibition of 35S rRNA production (72). Along these lines, a later study suggested that the immediate effects of rapamycin treatment on cellular 35S rRNA levels are likely due to rapid destabilization of rRNA precursors in response to reduced ribosomal protein levels rather than an instantaneous shut-down of Pol I transcription (61).

Our data confirm that in stationary *RPD3* and *rpd3*Δ cells Pol I transcription is strongly downregulated (Figures 2 and 3B) presumably as a long-term effect of TOR inhibition. However, we and others provide evidence for residual Pol I transcription in stationary *rpd3*Δ cells which is not observed in *RPD3* cells (9). The residual Pol I transcription in stationary *rpd3*Δ cells was required to maintain the open 35S rRNA gene chromatin state (Figure 6). The open chromatin state and residual Pol I transcription correlated with the recruitment of CF to 35S rDNA promoters because of increased protein levels of CF components in stationary *rpd3*Δ cells (Figures 3E and 5; Supplementary Figures S3C and S5). Increased protein levels were also observed for the Uaf30 and Rrn9 subunits of UAF in stationary *rpd3*Δ cells, but not for the UAF subunit Rrn5, Pol I subunits Rpa135 and Rpa190, Rrn3, or the yeast TBP, Spt15. It was not investigated if the absence of Rpd3 affects Rrn3-Pol I complex levels in stationary cells. In contrast to the reduced CF levels, reduced Uaf30 and Rrn9 levels in stationary *RPD3* cells did not significantly affect UAF association with 35S rDNA promoters or its nucleolar localization ((40), see Figures 3 and 4; Supplementary Figures S3 and S4). The observed differences might be due to different affinities of the respective protein complexes to their chromosomal binding sites. The latter notion is supported by previous results of *in vitro* template competition transcription assays (14,17,19).

How Rpd3 regulates the protein levels of CF and UAF components remains unclear. Deletion of *RPD3* resulted in a global up-regulation of the majority of endogenous Pol II transcripts in stationary cells (67). Along these lines, steady state level of mRNAs coding for CF and UAF components were increased in stationary *rpd3*Δ cells when compared to *RPD3* cells. Thus, enhanced transcription of the respective genes in the absence of Rpd3-mediated repression of Pol II transcription might explain the higher expression levels. On the other hand, the cellular protein levels of Rpa135, Rpa190, Rrn3, Rrn5 or Spt15, whose steady-state mRNA levels were also increased in stationary *rpd3*Δ cells (67), were not increased compared to *RPD3* cells (Figure 5 and Supplementary Figure S5). Differences in protein stabilities could be an explanation. There is the possibility that posttranslational covalent modification by acetylation—apart from modulating DNA-binding activities of UAF or CF—may influence protein stability. To our knowledge, no acetylation of CF or UAF specific subunits has yet been reported. Along these lines, Rpd3 may also play a role in regulating the degradation of nucleolar proteins by nucleophagy upon TOR inactivation (73). If UAF or CF components are subject to nucleophagy is not known.

### A role for CF in limiting the fraction of open rRNA genes

Taken together, our investigation of *RPD3* and *rpd3*Δ cells upon growth to the stationary phase suggests that Rpd3 affects Pol I transcription by adjusting the cellular level of CF components in stationary cells. The increased CF amounts augment the number of transcription-competent PICs at 35S rDNA promoters and the maintenance of the open rRNA gene chromatin state. In line with this interpretation, it is shown here that raising the endogenous level of CF components increases the number of open 35S rRNA genes in exponentially growing and stationary *RPD3* cells. This observation is also in accordance with the recent finding that increased endogenous CF levels may be a driver of Pol I transcription (74). The latter study reported increased rRNA synthesis in strains with enhanced expression of the genes coding for CF subunits *RRN7* and *RRN11*.

Regarding the physiological role of Rpd3 as a regulator of cell growth, Rpd3 was shown to be required to properly establish cellular quiescence in stationary yeast cells (67). In the absence of Rpd3 survival of yeast cells in the stationary phase is strongly diminished ((67), Supplementary Figure S4B). It is not known, if the maintenance of open rRNA gene chromatin contributes to this phenomenon. In another study it was shown that the abundance of rRNA genes is important for the maintenance of genome integrity (69). In the latter work, it was observed that yeast cells with a reduced rRNA gene copy number have an increased sensitivity towards the treatment with DNA-damaging drugs. It was concluded that the non-transcribed closed rRNA gene copies might be important to protect yeast cells from mutagens. Future experiments with the strains overexpressing CF proteins may help to find out if/how proper establishment of the closed chromatin state at the multicopy 35S rRNA gene loci supports cellular fitness and survival.

## Supplementary Material

gkae838_Supplemental_Files

## Data Availability

The data underlying this article are available in the article and in its online Supplementary Data.
