# Supplementary material for: Establishment of closed 35S ribosomal RNA gene chromatin in stationary Saccharomyces cerevisiae cells
Source: Nucleic Acids Res. 2024 Oct 7;52(20):12208–26. doi: 10.1093/nar/gkae838 (PMC11551728; doi:10.1093/nar/gkae838)
Supplement: gkae838_Supplemental_Files [file gkae838_supplemental_files.zip › Supplementary_data_070924.pdf]

SUPPLEMENTARY DATA

Supplemental Tables

Tables are organized as single worksheets within one MS Excel file.

Table S1:

Oligonucleotides used in this study. The respective oligonucleotide names/database numbers, sequences, as well as a short description are given. References (1–5).

Table S2:

Plasmids used in this study. The respective plasmid names/database number, details about plasmid construction, as well as a short description are given. References (1, 4, 6–10).

Table S3:

Yeast strains used in this study. For each strain, the name/database number, genotype, parental strain, and details about strain construction are given. The relevant Figures in which the results of experiments with the respective yeast strains are shown are listed. References (1, 2, 4, 5, 11–14).

Table S4:

Antibodies used in this study.

Table S5:

Templates used for Southern blot probe generation including details about template preparation and the target sequences. References (1, 4).

Table S6

Quantification of the fraction of open rRNA genes in Figures 1C, 2B, 6B, S6A, 7A,C. The fraction of open rRNA genes was calculated by dividing the pixel intensities of the band derived from open rRNA genes by the sum of the pixel intensities of the bands derived from open and closed rRNA genes.

Supplemental Datasets

Individual datasets are organized as single worksheets within one MS Excel file.

Supplemental Data Set SD1:

OD_{600} values of most of the cultures used in the experiments shown in Figures 1-7 and Supplemental Figure S2. In some Figures, the OD_{600} was additionally plotted against the time of growth after the exponential phase sample (0h) was withdrawn.

Supplemental Data Set SD2:

Raw data of ChIP- and RT-qPCR analyses shown in Figures 3B,C,D,E,F, 6C and S7. CT values for each sample are given. The fluorescence measurements plotted against the cycle number and melting curve analyses are shown as diagrams. Additionally, values for “take-off” and “amplification (=1+E)” which are used by the comparative quantitation module of the RotorGene Q software to compute the “comparative concentration” used to calculate the fraction of immuno-precipitated DNA or normalized cDNA quantity are shown.

Supplemental Figure Legends

Supplemental Figure S1 related to Figures 1 and 2

Schematic representation of psoralen crosslinking, Chromatin Endogenous Cleavage (ChEC), and a combination of ChEC with psoralen crosslinking analyses

A) Psoralen crosslinking distinguishes between open and closed 35S rRNA gene chromatin states. Cells are incubated in the presence of psoralen (depicted as X). Psoralen intercalates into genomic DNA but intercalation is prevented by nucleosomes (depicted as cylinders). Upon irradiation with long-wave UV light, psoralen forms a covalent bond between the two DNA single strands. DNA is isolated, cleaved with restriction enzymes (restriction sites depicted as arrows), separated in a native agarose gel, and analyzed in a Southern blot. The degree of psoralen incorporation determines the mobility of DNA fragments in the agarose gel. If a genomic region exists in two different chromatin states, e.g. nucleosomal “closed” and nucleosome depleted “open”, two populations of DNA fragments differing in their degree of psoralen incorporation (and thus in their migration in the gel) are obtained.

B) ChEC determines the association of Micrococcus Nuclease (MN) fusion proteins with genomic loci. Cells expressing a protein of interest (POI) in fusion with MN (depicted as “packman”) are subjected to formaldehyde crosslinking covalently fixing chromosomal proteins to their DNA-association sites. After the preparation of crude nuclei, calcium addition activates the MN moiety of the fusion proteins. Activated MN cleaves the DNA in the proximity of the respective binding sites. DNA is isolated cleaved with restriction enzymes (restriction sites depicted as arrows) separated in a native agarose gel and analyzed in a Southern blot to reveal MN fusion protein-specific cleavage events. Note that cleavage depends on DNA accessibility in the chromatin context and the DNA sequence preference of the MN.

C) The combination of ChEC with psoralen crosslinking detects the specific association of a POI with the open or the closed 35S rRNA gene chromatin state. If ChEC is performed in a strain expressing an MN fusion protein of a POI which is selectively associated with the open chromatin state, specifically the strongly psoralen-crosslinked DNA fragment derived from the open chromatin state will be degraded.

Supplemental Figure S2 related to Figure 2

Open rRNA genes in stationary rpd3Δ cells are largely devoid of Pol I and are bound by Hmo1

RPD3 or *rpd3*Δ strains y1717, y2256, y1761, and y2257, expressing Rpa190^{MN} or Hmo1^{MN} were grown in YPAD at 30°C to exponential phase (exp) or for another 144h to stationary phase (stat). Samples were withdrawn at each time point and either mock-treated (0min ChEC) or subjected to ChEC experiments for different time points indicated on the top, as described in the legend of Figure 2. A, C). Autoradiograms were analyzed by plotting the normalized radioactive intensities in the lanes after 60min ChEC in samples from *RPD3* (grey lines) and *rpd3*Δ cells (dotted black lines; graphs on the right of the autoradiograms). The intensities were normalized to the intensity of the undigested full-length fragment (labeled by an arrow). A map of the XcmI fragment under investigation is depicted on the right of the graphs (genetic elements as explained in Figure 1A). B, D) The percentage of

degradation of the full-length XcmI fragment was calculated by dividing the background-corrected integrated radioactive intensity of cleavage products by the sum of the integrated radioactive intensity of cleavage products and the integrated radioactive intensity of the full-length XcmI fragment for each lane. The bar graphs show the mean values for the three different time points of ChEC. The error bars represent the respective standard deviations. Representative results of two (Figure S2A and B) and three (Figure S2C and D) biological replicates are shown.

Supplemental Figure S3 related to Figure 3

RPD3 deletion affects Pol I PIC formation in the stationary phase

RPD3 or *rpd3*Δ strains y1151, y3028, y1185, y3030, y881, and y3024, expressing Rrn9^{MN} (A), Spt15^{MN} (B), and Rrn7^{MN} (C), were grown in YPAD at 30°C to exponential phase (exp), or for another 144h to stationary phase (stat), and samples were withdrawn. Samples were either mock-treated (0min ChEC) or subjected to ChEC experiments and subsequent Southern blot analysis as described in the legend of Figure 2A. Representative results of three (Figure S3A and C) and two (Figure S3B) biological replicates are shown.

Supplemental Figure S4 related to Figure 4

A, B) Deletion of RPD3 significantly increases the number of lysed cells in the stationary phase

RPD3 or *rpd3*Δ strains, K699, y3678 were grown in YPAD+ at 30°C to exponential phase (exp) or for another 129h to stationary phase (stat) before samples were withdrawn and subjected to live-cell fluorescence microscopy. A) Micrographs after differential interference contrast (DIC) imaging, and fluorescence imaging using the channel for detection of 3mCherry or GFP signals are depicted. Red arrows mark “lysed” cells. B) A population of 100 cells was classified into “intact” or “lysed” cells as judged by the appearance in DIC images. The cell count is depicted in bar graphs as the percentage of the total population. Representative results of two biological replicates are shown.

C-H) RPD3 deletion increases nucleolar levels of CF subunits in stationary cells

RPD3 or *rpd3*Δ strains W17019, W17020, W17023, W17024, W17343, and W17344, expressing Hmo1^{3mCherry} and either Rrn5^{GFP}, Uaf30^{GFP} or Rrn6^{GFP} were grown in YPAD+ at 30°C to exponential phase or for another 129h to stationary phase before samples were withdrawn and subjected to live-cell fluorescence microscopy. C, E, G) Micrographs after differential interference contrast (DIC) imaging, and fluorescence imaging of 3mCherry or GFP are depicted. In merge images, GFP is depicted in cyan and 3mCherry in red. Red and green arrows point to lysed cells and cells classified to have “adjacent” fluorescent signals, respectively (see below). D, F, H) A population of 100 cells was classified into four different cellular phenotypes according to the observed fluorescence as “adjacent” signals, “colocalization” of GFP and 3mCherry, “no nucleolar GFP”, or judged by the appearance in DIC images as “lysed”. The cell count for each phenotype is depicted in bar graphs as the percentage of the total population. Color coding of the bar graphs is depicted in D). Statistical analyses are described in the Materials and Methods section. Representative results of two biological replicates are shown.

Supplemental Figure S5 related to Figure 5

RPD3 deletion increases protein levels of UAF and CF components in stationary cells

A) Images of the full membranes after Ponceau S (Ponceau) staining of the membranes as well as chemiluminescence signals after detection of TAP-fusion proteins with Peroxidase Anti-Peroxidase Soluble Complex (α-PAP) shown in Figure 5A. Additionally, the membranes were re-probed with anti-tubulin antibodies (α-tubulin) followed by secondary peroxidase coupled anti-rat IgG antibodies. The position of the yeast protein Tub1 recognized by the antibody is indicated on the right. Residual signals from Peroxidase Anti-Peroxidase Soluble Complex (α-PAP) bound to TAP-fusion proteins are labeled by asterisks. Positions of selected marker proteins are indicated by their molecular weight in kDa on the left side of the blot images. Representative results of two biological replicates are shown.

B) *RPD3* (WT) or *rpd3*Δ (Δ) strains, W17017, W17018, W17019, W17020, W17021, W17022, W17023, W17024, W17339, W17341, W17343, and W17344, expressing Hmo1^{3mCherry} and either Rpa190^{GFP}, Uaf30^{GFP}, Rrn5^{GFP}, Rrn9^{GFP}, Rrn6^{GFP} or Rrn7^{GFP} were grown in YPAD+ at 30°C to exponential phase or for another 129h to stationary phase before samples were withdrawn. As a control *RPD3* (WT) or *rpd3*Δ (Δ) strains W10878, W10879, W16791, and W16792 expressing Hmo1^{3mCherry} but no GFP-fusion protein were cultured and treated in parallel. Whole-cell extracts were prepared and analyzed in a western blot. After transfer to the membrane proteins were visualized by staining with Ponceau S (Ponceau). GFP-fusion proteins were detected with anti-GFP antibodies (α-GFP), followed by fluorescently labelled secondary anti mouse IgG antibodies. Additionally, the membranes were re-probed with anti-tubulin antibodies (β-tubulin) followed by secondary fluorescently labelled anti-rabbit IgG antibodies. Whole-cell extracts from the same number of cells were loaded for each sample set derived from strains expressing a certain GFP-fusion protein. Images of fluorescent signals as well as Ponceau S staining of the full membranes are shown. The positions of bands corresponding to the respective full-length GFP fusion protein are labelled with a triangle, the position of bands corresponding to tubulin are labelled by an asterisk. Positions of selected marker proteins are indicated by their molecular weight in kilodalton (kDa) on the left side of the blot images. Representative results of two biological replicates are shown.

Supplemental Figure S6 related to Figure 6

Psoralen accessibility of yeast 35S rRNA genes is not significantly influenced by temperature shift to 37°C

RPD3 wild-type strain NOY505 or *rpd3*Δ strains y2919 and 2614 carrying an *HMO1* wild-type allele, and a complete deletion of the gene (*hmo1*Δ), respectively were grown in YPAD at 24°C to exponential phase (exp), or for another 144h to stationary phase (stat), and samples were withdrawn (0h). At both time points, a fraction of the culture was shifted to 37°C, and samples were withdrawn after the indicated times of incubation. All samples were subjected to psoralen photo-crosslinking analyses. DNA was isolated, digested with EcoRI and analyzed in a Southern blot as described in the legend of Figure 2B. Autoradiograms show results for the 2.9kb 25S rDNA fragment. The positions of strongly psoralen-crosslinked fragments derived from “open” rRNA genes and of weakly psoralen-

crosslinked “closed” rRNA genes are indicated on the right. Graphs on the bottom show analyses of the radioactivity profile in the lanes with samples of cells cultured at 24°C (0h) (dotted black lines), and of cells after 5h incubation at 37°C (grey lines). The positions of the peaks corresponding to the fragments derived from open (o) and closed (c) rRNA genes are labeled on the x-axis of the graphs at the bottom. Representative results of two biological replicates are shown.

Supplemental Figure S7 related to Figure 7

Genomically integrated expression cassettes increase endogenous mRNA levels of CF genes.

RPD3 strains NOY505, y4966, y4967, y4969, and y4970 expressing CF genes from the endogenous locus, and carrying a genomically integrated expression cassette for CF genes under the control of a p*SPT15* or p*TEF1* promoter (p*SPT15-CF*, p*TEF1-CF*) were grown in YPAD at 30°C to exponential phase (exp). RNA was isolated and subjected to RT-qPCR analysis using primer pairs detecting the cDNAs from *RRN6*, *RRN7*, *RRN11*, and *UAF30*, and *PDC1*. The amount of *RRN6*, *RRN7*, *RRN11*, and *UAF30* cDNA relative to *PDC1* cDNA was determined for each sample. The bar graph depicts the relative RNA amounts in strains y4966 (clone 1), y4967 (clone 2), y4969 (clone 1), and y4970 (clone 2) normalized to the relative RNA amounts in strain NOY505. The mean and standard deviation were derived from triplicate qPCR reactions. Representative results of two biological replicates are shown.

Supplemental References (included in Supplemental Tables)

1. Merz,K., Hondele,M., Goetze,H., Gmelch,K., Stoeckl,U. and Griesenbeck,J. (2008) Actively transcribed rRNA genes in S. cerevisiae are organized in a specialized chromatin associated with the high-mobility group protein Hmo1 and are largely devoid of histone molecules. Genes Dev., 22, 1190–1204.

2. Goetze,H., Wittner,M., Hamperl,S., Hondele,M., Merz,K., Stoeckl,U. and Griesenbeck,J. (2010) Alternative chromatin structures of the 35S rRNA genes in Saccharomyces cerevisiae provide a molecular basis for the selective recruitment of RNA polymerases I and II. Mol. Cell. Biol., 30, 2028–2045.

3. Hierlmeier,T., Merl,J., Sauert,M., Perez-Fernandez,J., Schultz,P., Bruckmann,A., Hamperl,S., Ohmayer,U., Rachel,R., Jacob,A., et al. (2013) Rrp5p, Noc1p and Noc2p form a protein module which is part of early large ribosomal subunit precursors in S. cerevisiae. Nucleic Acids Res., 41, 1191–1210.

4. Wittner,M., Hamperl,S., Stöckl,U., Seufert,W., Tschochner,H., Milkereit,P. and Griesenbeck,J. (2011) Establishment and maintenance of alternative chromatin states at a multicopy gene locus. Cell, 145, 543–554.

5. Girke,P. and Seufert,W. (2019) Compositional reorganization of the nucleolus in budding yeast mitosis. Mol. Biol. Cell, 30, 591–606.

6. Wai,H.H., Vu,L., Oakes,M. and Nomura,M. (2000) Complete deletion of yeast chromosomal rDNA repeats and integration of a new rDNA repeat: use of rDNA deletion strains for functional analysis of rDNA promoter elements in vivo. Nucleic Acids Res., 28, 3524–3534.

7. Griesenbeck,J., Boeger,H., Strattan,J.S. and Kornberg,R.D. (2004) Purification of defined chromosomal domains. Methods Enzymol., 375, 170–178.

8. Puig,O., Caspary,F., Rigaut,G., Rutz,B., Bouveret,E., Bragado-Nilsson,E., Wilm,M. and Séraphin,B. (2001) The tandem affinity purification (TAP) method: a general procedure of protein complex purification. Methods San Diego Calif, 24, 218–229.

9. Sheff,M.A. and Thorn,K.S. (2004) Optimized cassettes for fluorescent protein tagging in Saccharomyces cerevisiae. Yeast Chichester Engl., 21, 661–670.

10. Janke,C., Magiera,M.M., Rathfelder,N., Taxis,C., Reber,S., Maekawa,H., Moreno-Borchart,A., Doenges,G., Schwob,E., Schiebel,E., et al. (2004) A versatile toolbox for PCR-based tagging of yeast genes: new fluorescent proteins, more markers and promoter substitution cassettes. Yeast Chichester Engl., 21, 947–962.

11. Nogi,Y., Yano,R., Dodd,J., Carles,C. and Nomura,M. (1993) Gene RRN4 in Saccharomyces cerevisiae encodes the A12.2 subunit of RNA polymerase I and is essential only at high temperatures. Mol. Cell. Biol., 13, 114–122.

12. Claypool,J.A., French,S.L., Johzuka,K., Eliason,K., Vu,L., Dodd,J.A., Beyer,A.L. and Nomura,M. (2004) Tor pathway regulates Rrn3p-dependent recruitment of yeast RNA polymerase I to the promoter but does not participate in alteration of the number of active genes. Mol. Biol. Cell, 15, 946–956.

13. Brachmann,C.B., Davies,A., Cost,G.J., Caputo,E., Li,J., Hieter,P. and Boeke,J.D. (1998) Designer deletion strains derived from Saccharomyces cerevisiae S288C: a useful set of strains and plasmids for PCR-mediated gene disruption and other applications. Yeast Chichester Engl., 14, 115–132.

14. Hannig,K., Babl,V., Hergert,K., Maier,A., Pilsl,M., Schächner,C., Stöckl,U., Milkereit,P., Tschochner,H., Seufert,W., et al. (2019) The C-terminal region of Net1 is an activator of RNA polymerase I transcription with conserved features from yeast to human. PLoS Genet., 15, e1008006.

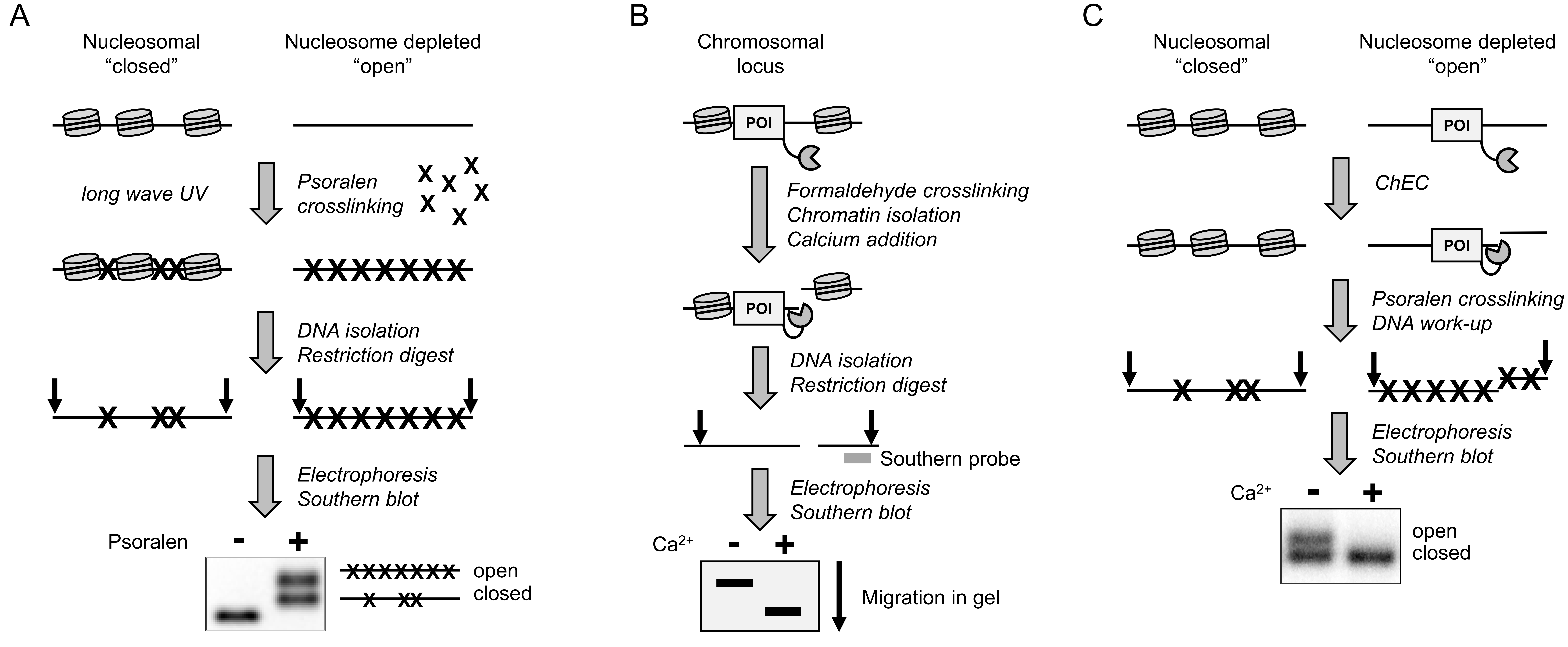

Babl Fig. S1 related to Fig. 1 & 2

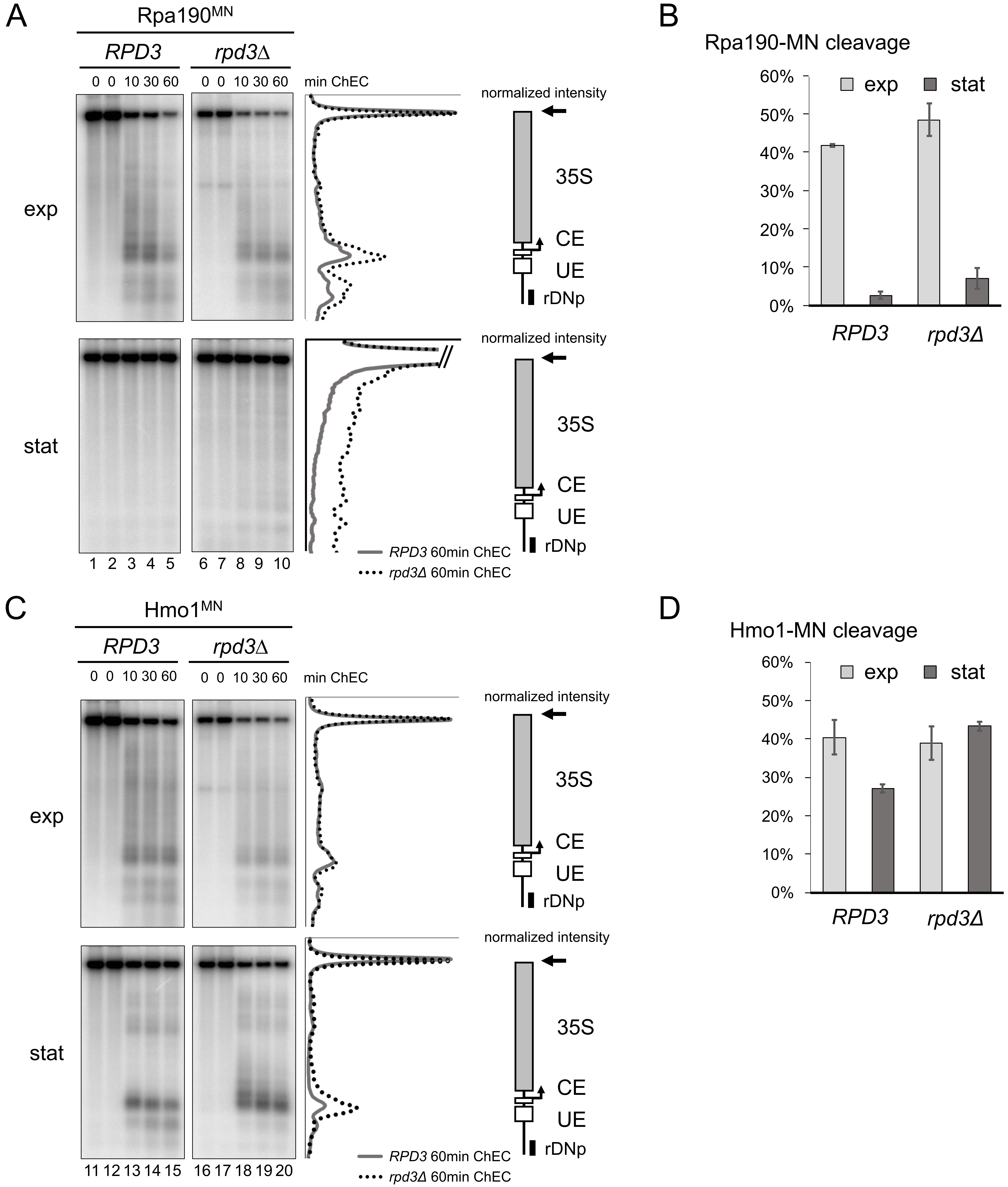

Babl Fig. S2 related to Fig. 2

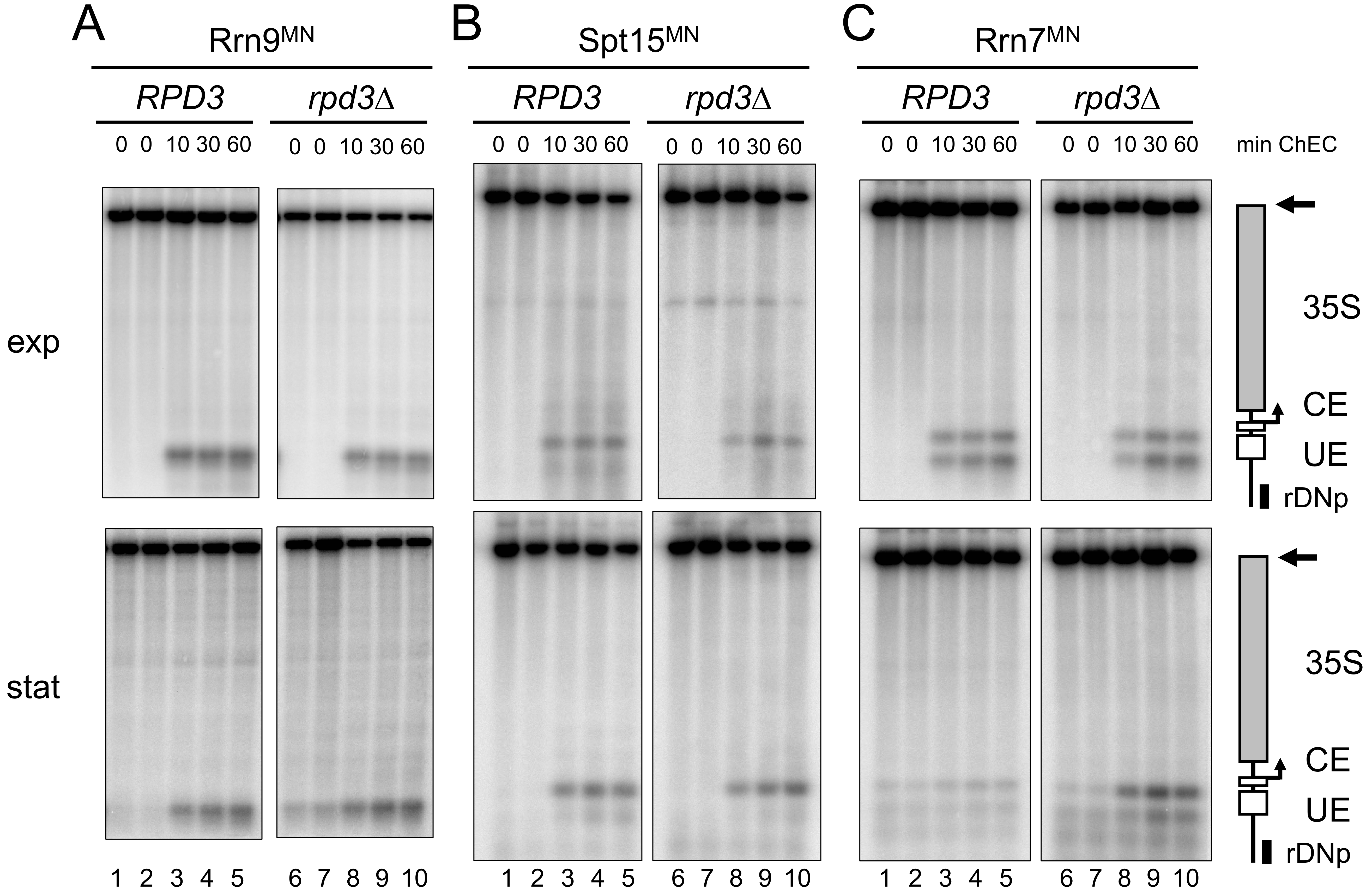

Babl Fig. S3 related to Fig. 3

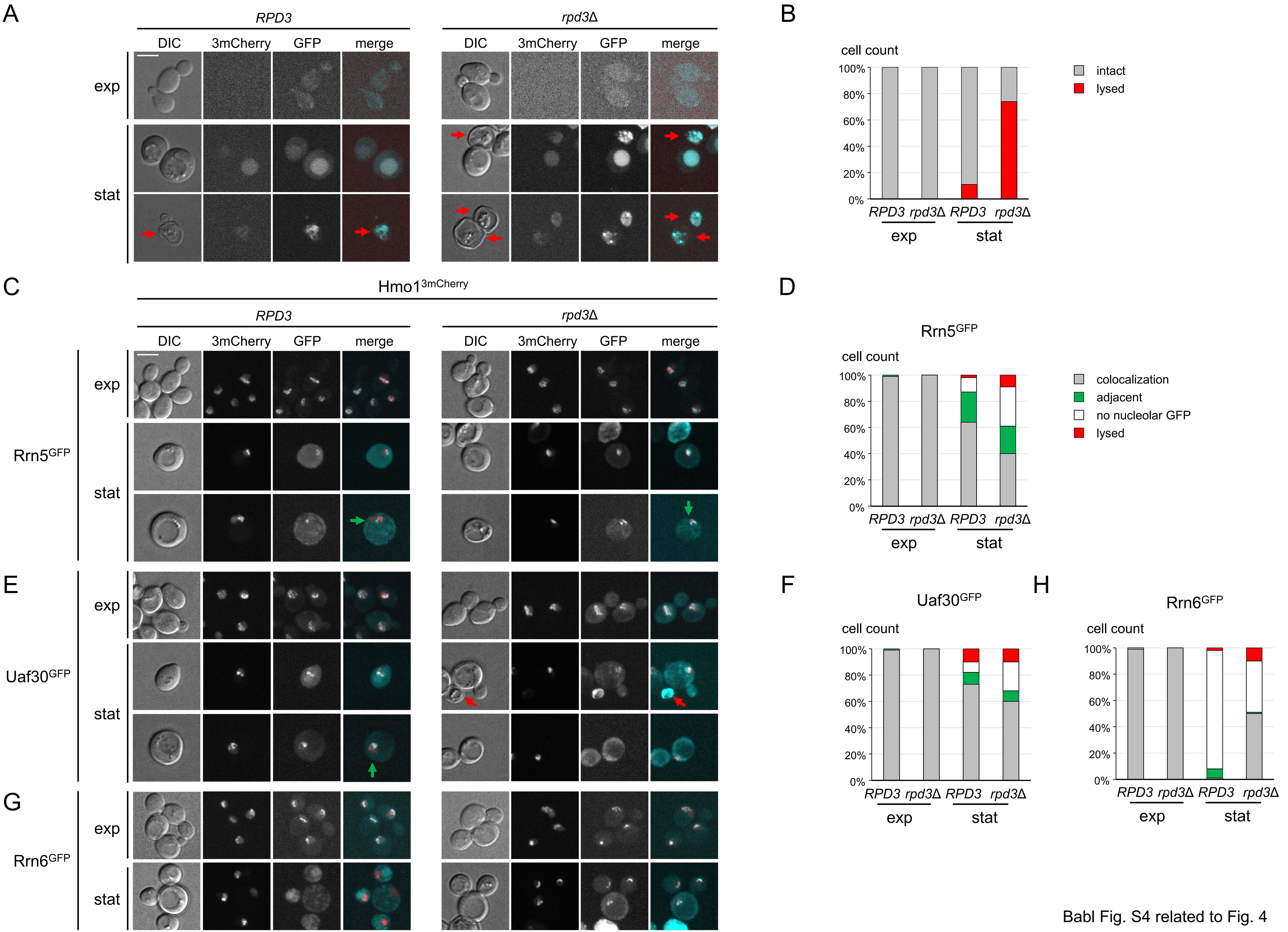

Babl Fig. S4 related to Fig. 4

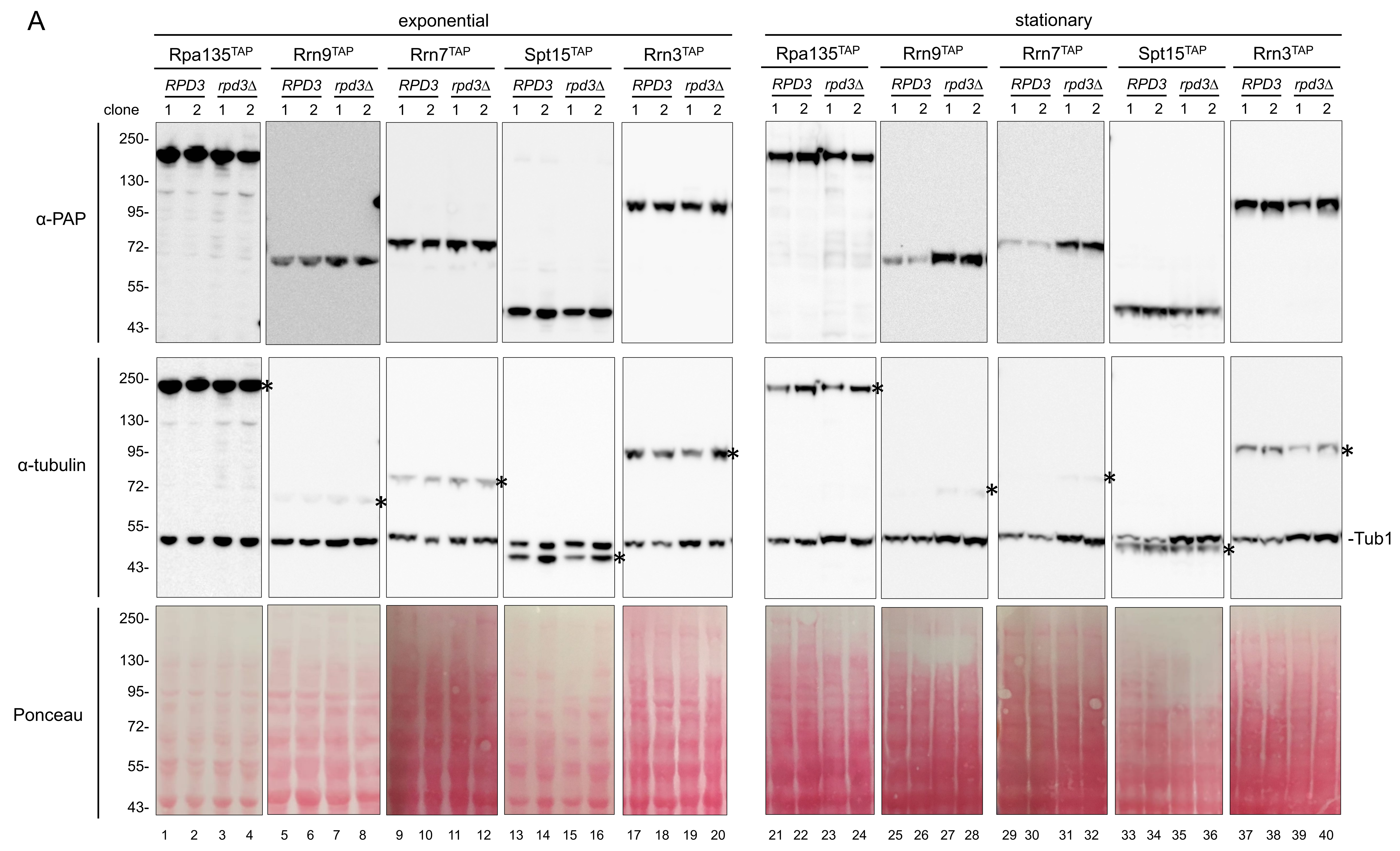

Babl Fig. S5A related to Fig.5

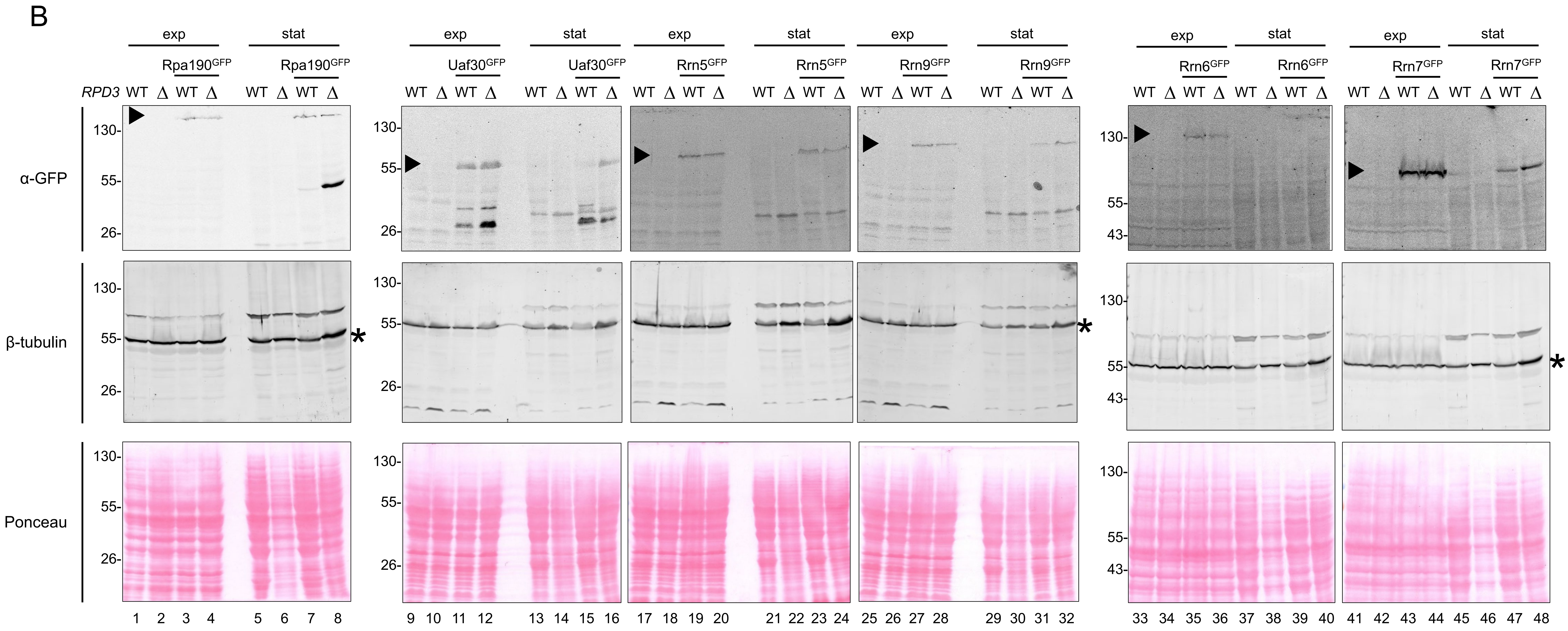

Babl Fig. S5B related to Fig.5

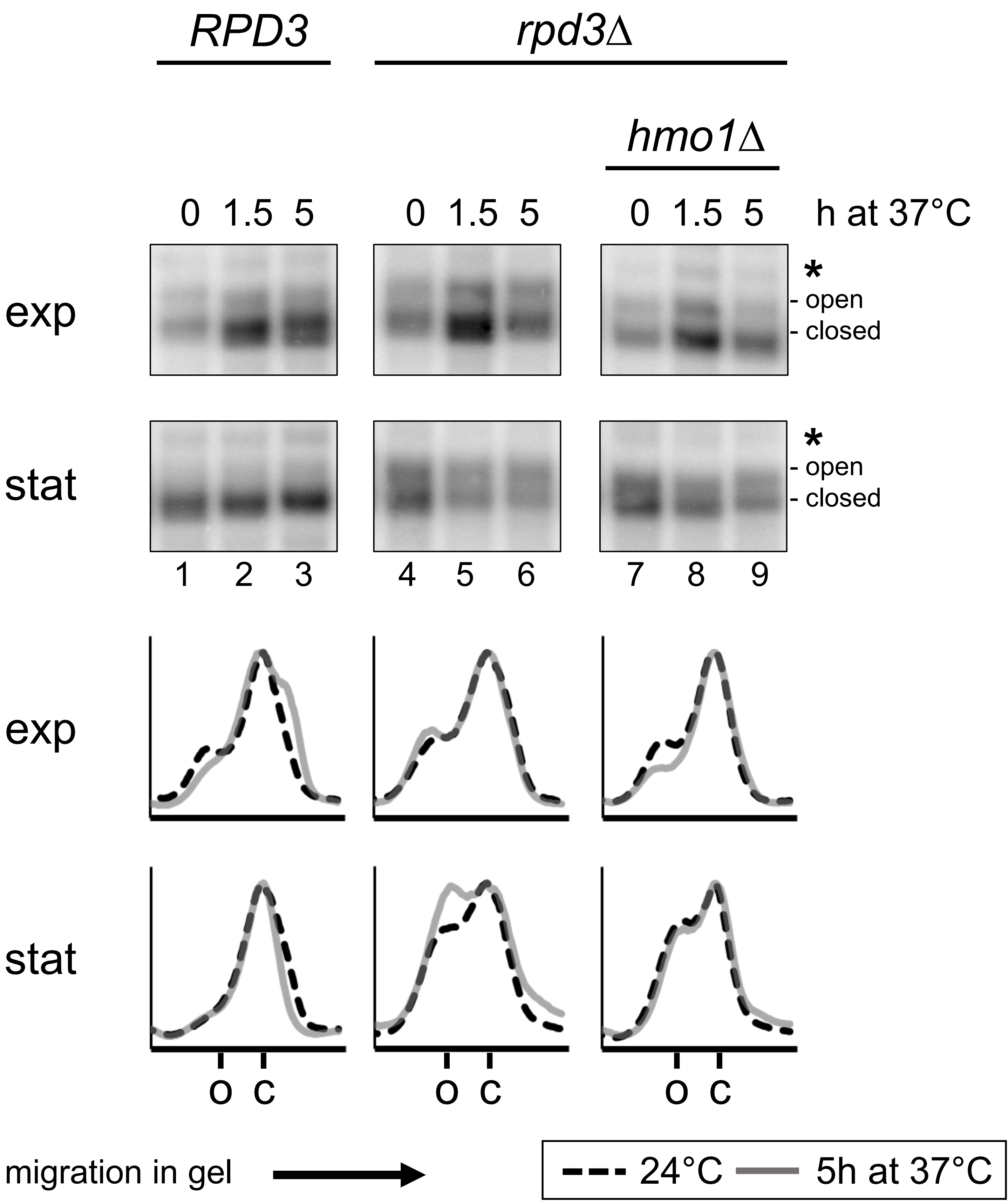

Babl Fig. S6 related to Fig. 6

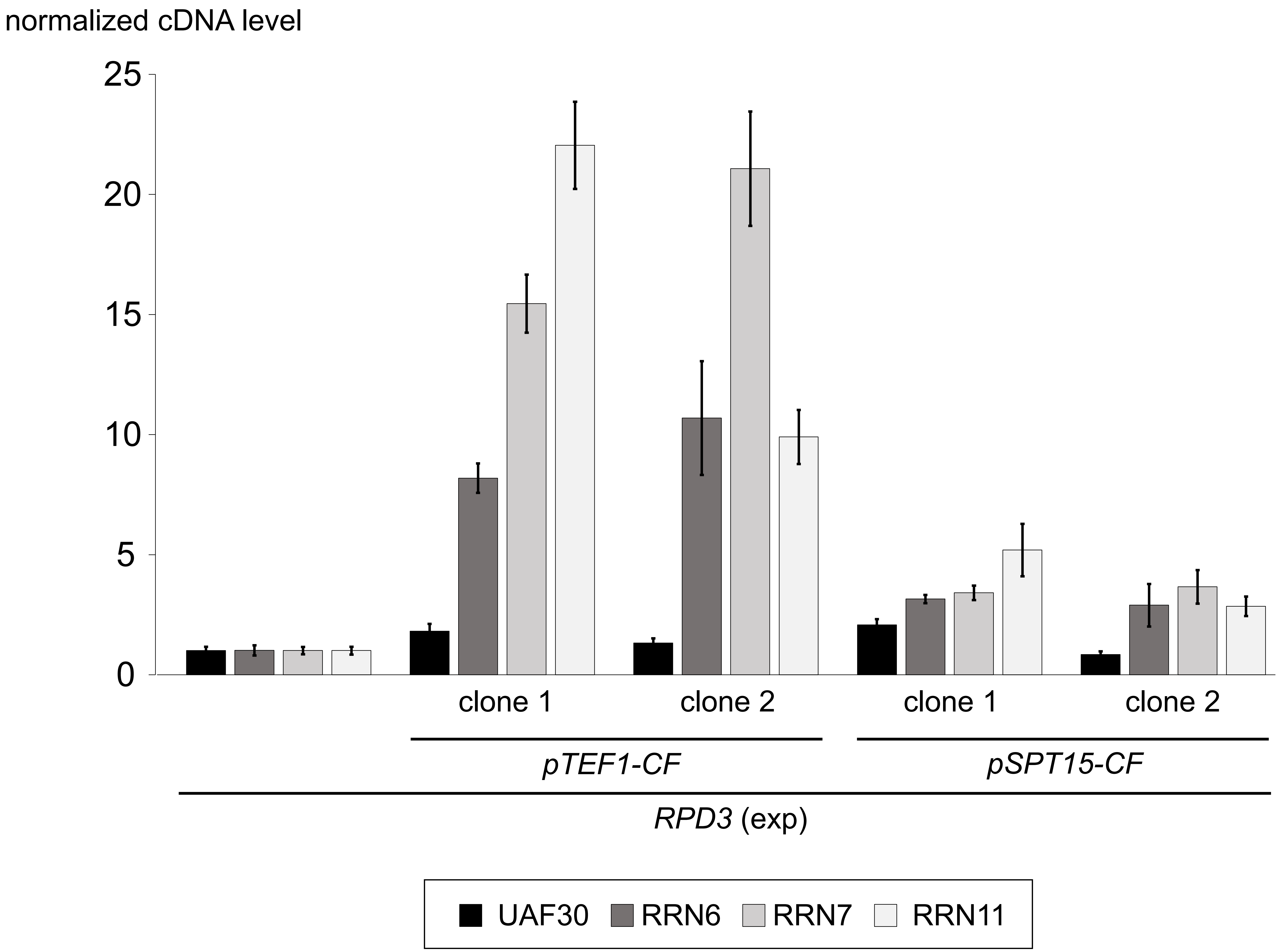

Babl Fig. S7 related to Fig. 7
